# Genetic Structure and Signs of Local Adaptation in Icelandic Brown Trout Populations

**DOI:** 10.1002/ece3.74274

**Published:** 2026-09-11

**Authors:** Marcos Lagunas, Arnar Pálsson, Lea Jerman‐Plesec, Zophonías O. Jónsson, Sigurður S. Snorrason

**Affiliations:** ^1^ Institute of Life and Environmental Sciences University of Iceland Reykjavík Iceland; ^2^ Department of Cellular and Molecular Medicine, Faculty of Health and Medical Sciences University of Copenhagen Copenhagen Denmark

**Keywords:** anadromy, brown trout, ddRADseq, Iceland, isolation, SNPs

## Abstract

Loss of anadromy in Salmonids frequently follows postglacial colonization and habitat change, a process driven by selective pressures where benefits of residency outweigh migration costs. At the end of the last Ice Age *ca*. 10,000 years ago, anadromous brown trout arrived in Iceland and invaded newly formed habitats. Some populations became isolated in headwaters early after the ice retreated, presumably accelerating local adaptation and the evolution of residency. In this study, we assessed the genetic relatedness and diversity of brown trout on a countrywide scale to decipher the number of unique populations and their connectivity. We tested for gene flow from headwaters and assessed effects of isolation and adaptation. We genotyped 600 individuals from 40 locations using ddRADseq, identifying 2946 putative neutral SNPs. We identified 16 groups of genetically similar individuals (genetic clusters), five of which were primarily composed of anadromous individuals. On a large scale, the genetic variation and relatedness of trout populations reflect the geography of waterways while within watersheds the topography, for example, the presence of waterfalls and lakes, is key. Counterintuitively, downstream gene flow from isolated headwater populations was only seen in small river populations whereas gene flow from large lake populations appears negligible. Using outlier SNPs, we identified several hundred loci associated with temperature and habitat type (i.e., lake vs. river). Temperature‐associated markers mapped to loci associated with immune response, lipid metabolism and transmembrane protein genes. Our results highlight the effects of isolation on genetic diversity in headwater populations and the importance of identifying unique populations in conservation efforts.

## Graphical abstract

We present here an assessment of the genetic structure of Icelandic brown trout across 40 locations, revealing 16 distinct genetic clusters shaped by postglacial isolation and local adaptation. Our research highlights that landlocked headwater populations exhibit significantly reduced genetic diversity compared to anadromous groups and identifies specific genomic markers associated with temperature adaptation and the loss of migratory behavior. Additionally, the findings demonstrate how anthropogenic stocking in a lake system altered natural genetic patterns, highlighting the need to protect unique, isolated populations for conservation.

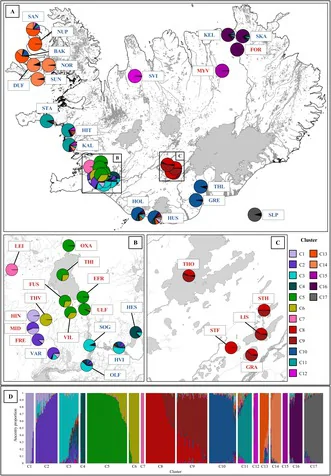

## 1 Introduction

Many salmonid species have both anadromous (spawning in rivers, feeding, and maturing at sea) and resident freshwater populations. While the origin of diadromy in salmonids is debated (McDowall 2002), it is generally assumed that resident populations within these species represent descendants of anadromous ancestors, an assumption that seems logical for salmonids that invaded newly formed watersheds at the end of the last glacial epoch (Bernatchez 2001; Hamilton et al. 1989). Theoretically, the maintenance of anadromy or evolution of residency would depend on the balance between costs and benefits of the alternative strategy (Hendry et al. 2004). Anadromy incurs substantial energetic costs for individuals, including long‐distance migration to sea and back to spawning sites, physiological stress during smoltification and osmoregulation transitions between freshwater and seawater (McCormick and Saunders 1987), and elevated mortality risks from marine predators. These costs may be offset by benefits such as accelerated growth in the marine environment and higher fecundity associated with larger body sizes. Conversely, residency avoids migration risks and physiological challenges but typically constrains growth potential and reproductive output in smaller headwater habitats. It is logical to assume that in anadromous populations the costs are on average outweighed by the benefits.

Changes to waterways, such as the formation of unscalable waterfalls or man‐made obstacles to migration, cause fundamental changes to anadromous populations that spawn above such impasses. Postglacial waterways of the northern hemisphere harbor numerous salmonid populations in tributaries and lakes that are isolated by unscalable waterfalls (Pitman et al. 2020; Subramanian and Kumar 2023; Waples et al. 2008), demonstrating their flexibility in life history and genetic variation to avert extinction. Here, we use “headwaters” to refer to the upper reaches of river systems: streams and lakes situated upstream in watersheds, often at higher elevation and frequently isolated from downstream reaches by impassable waterfalls. When natural barriers such as waterfalls have formed, the return of spawners from the sea becomes more difficult or impossible, thus favoring resident phenotypes over migratory ones. For example, we would expect selection for earlier maturity, reduced migratory behavior, and altered osmoregulatory capacity, as migration becomes costly or impossible (Hendry et al. 2004). This directional selection, together with genetic drift in small isolated populations, may have played a prominent role in promoting the diversification seen in some salmonid species (Subramanian and Kumar 2023; Waters et al. 2020).

Environmental changes that modify selective pressures will alter allele frequencies (Reed et al. 2011), and the degree to which both anadromous and resident populations will experience this depends on their genetic similarity and the environmental change (Kjaerner‐Semb et al. 2021). Studies on Arctic charr ( *Salvelinus alpinus* ) suggest limited genetic parallelism in loss of anadromy (Salisbury et al. 2020), while in rainbow trout ( *Oncorhynchus mykiss* ), specific genomic regions associate repeatedly with lake adaptations (Pearse et al. 2019). Research on patterns of genetic differentiation in other salmonid species is needed to shed more light on genomic responses to environmental change, as are tests for association of specific markers with variation or change in habitats and temperature regimes.

Iceland sits on the Mid‐Atlantic Ridge and has experienced frequent volcanism and isostatic movements following the last glacial epoch (Sigmundsson 1991). Around 10,000 ^14^C years BP the sea level was *ca*. 40 m above the current mark (Norðdahl et al. 2008), likely facilitating colonization by anadromous brown trout and Arctic charr ( *Salvelinus alpinus* ) as watersheds became ice‐free. Subsequent isostatic rebound, diminished flow of glacial rivers, tectonic activity, and frequent volcanic activity created impassable waterfalls that isolated populations of Arctic charr (Kapralova et al. 2011) and brown trout in headwaters (Lagunas et al. 2023; Sturlaugsson and Malmquist 2011). Some of the topographic changes were abrupt and may have caused local extinctions or severe population bottlenecks. Two such headwater systems are in focus in this study: (a) the Veiðivötn lake swarm and Lake Þórisvatn in the southern highlands, and (b) Lakes Þingvallavatn and Úlfljótsvatn in the south‐west (see Section 2.1 for detailed descriptions).

Despite low mitochondrial DNA diversity (Bernatchez 2001; Hynes et al. 1996; Pálsson and Árnason 1994), Icelandic brown trout show extensive phenotypic variation. Fish from spring‐fed rivers with heterogeneous feeding grounds that reduce competition exhibit more rapid juvenile growth and mature earlier than fish from more homogeneous, run off rivers (Antonsson and Jóhannsson 2012). In the Hengill mountain, brown trout inhabit streams, both cold and influenced by geothermal heat (temperature range 4°C–20°C). In warm water they are more selective and feed on higher trophic levels of invertebrates (e.g., predatory dipteran larvae) (O'Gorman et al. 2016). In contrast, where temperatures are lower (~4°C), they feed on primary consumers (e.g., snails). This may not only reflect a change in the community structure of invertebrates, but also their need for more energy‐rich sources of food when their metabolism is higher due to warmer environmental temperatures (O'Gorman et al. 2016). Importantly, the Hengill brown trout reach sexual maturity at a small size (i.e., max length up to 30 cm) and are therefore referred to as dwarves (Ólafsson 2019).

Single nucleotide polymorphisms have previously been used to infer the genetic structure of brown trout populations in local geographic scales (e.g., Lagunas et al. 2023; Lemopoulos et al. 2019; Massa‐Gallucci et al. 2010; Saha et al. 2022) but less often at larger regional scales, for example, Bekkevold et al. (2020). In this study we use genome‐wide SNPs from ddRADseq data to assess the genetic variation and differentiation among Icelandic brown trout populations on a countrywide scale. In doing so, we aim to cast light on some core aspects with broad relevance to evolutionary biology and conservation: (i) how geography and the physical evolution of waterways (e.g., formation of waterfalls) have shaped the postglacial evolution and differentiation among populations of brown in Iceland, (ii) how isolation of populations can cause loss of genetic variation via genetic drift and local adaptation, and (iii) we will look for genomic signatures of local adaptation to environmental gradients (temperature, lake vs. river habitat) that may be used to predict how salmonids may respond to future climate change.

Iceland provides a unique natural environment to address these questions due to its young postglacial age, geological history, and presence of both anadromous and resident brown trout populations. As seen in other studies (González‐Ferreras et al. 2022; Leclerc et al. 2008) we expected to find a pattern of genetic relatedness reflecting geography and watershed geomorphology, for example, the effect of waterfalls on direction of gene flow. Several trout populations have long been isolated above unscalable waterfalls (e.g., populations of Lake Þingvallavatn and lakes in the Veiðivötn area). As they may have evolved independently for a long time, we expected them to show lower genetic diversity and higher differentiation. The adaptation to local environmental factors and the switch from anadromous to residential lifestyle may have influenced allele frequencies at specific loci. We investigated the genomic signatures of local adaptation by testing for associations between allele frequencies and environmental variables (i.e., summer temperature regimes) and habitat type (i.e., lake vs. river). We also examined patterns of genomic divergence between anadromous and isolated headwater populations to identify loci linked to the loss of migratory behavior. While our focus is on Icelandic brown trout, the conceptual framework applies to any salmonid populations where colonization history, landscape fragmentation, and environmental heterogeneity interact to shape genetic diversity and adaptive potential.

## 2 Materials and Methods

### 2.1 Study Area and Sample Collection

Icelandic freshwater systems are young, having formed since the last glacial maximum, and the postglacial history is marked by isostatic rebound, volcanic activity, and tectonic dynamics that have repeatedly modified the topography of waterways. Two headwater systems are of particular interest in our study: (i) the Veiðivötn lake swarm and Lake Þórisvatn in the southern highlands, and (ii) Lakes Þingvallavatn and Úlfljótsvatn in the south‐west.

The Veiðivötn system consists of *ca*. 23 lakes (most of them < 1 km^2^) and several small ponds at 560–600 m above sea level. At present some of these lakes are landlocked, with no visible runoff, but some drain to a small river, Vatnakvísl, that joins the glacial river Tungnaá, a major tributary of Þjórsá. The area has experienced at least 15 volcanic eruptions since the last glacial maximum (Larsen 1984, 2023). Two of these eruptions (in 877 and 1477 AD) started with an explosive phase, spewing huge amounts of tephra and temporarily damming the River Tungnaá, thus forming a large lake, Langalón (*ca*. 140 km^2^), extending to the north in the Veiðivötn depression and southwards in the area south of the river (Larsen 1984, 2023). The damming likely formed multiple refuges in the southern and eastern parts of Langalón, where trout could escape from the heaviest downpour of tephra. The lakes in Veiðivötn, that had open access to Tungnaá, were renowned for excellent trout fishery and for ages they were utilized by farmers (Jóhannsson et al. 2002). In the 20th century, when the area became accessible by motor vehicles, farmers started stocking the landlocked lakes in Veiðivötn and Þórisvatn. One drawback was the apparent lack of suitable spawning sites which demanded regular restocking (Jóhannsson et al. 2002). The first documented stocking was done by releasing a few adult fish from Stóra Fossvatn, the only lake in Veiðivötn that is isolated at present by a waterfall but with well‐known spawning grounds, in Þórisvatn (25 trout) and in two landlocked lakes, Litlisjór (19 trout) and Grænavatn (14 trout) (Jónsson 1951). In the 1960's an effort was started in stocking reared trout fingerlings in many of the lakes in the Veiðivötn system, first using the spawners in Stóra Fossvatn and later mature fish caught in Litlisjór as broodstock (Jóhannsson 2017).

The Þingvallavatn/Úlfljótsvatn system, shaped by the interplay of tectonic rifting and recurrent volcanism, comprises two interconnected lakes. Lake Þingvallavatn (83 km^2^) with a mean depth of 34 m, lies *ca*. 100 m above sea level and discharges into the much smaller lake Úlfljótsvatn (2.3 km^2^). Around 10,000 ^14^C years BP a small glacial lake was formed which grew larger as the ice receded (Saemundsson 1992). Following further retreat of the ice and isostatic rebound (Ingólfsson et al. 1995), volcanic eruptions altered inflowing water sources, stabilizing the flow of groundwater seeping through the lava via underground fissures (Adalsteinsson et al. 1992). This stabilized the lake's discharge, creating ecological conditions that would enhance the evolution of residency. Currently, two impassable waterfalls below Úlfljótsvatn, Írafoss and Kistufoss, at ~60 and ~50 m above sea level, block access to the lakes. Our prior study using ddRADseq revealed that Þingvallavatn hosts a metapopulation of brown trout, presently dominated by a genetic component from the major spawning grounds in River Öxará, the main river draining into the lake (Lagunas et al. 2023). We also observed that gene flow from an isolated population inhabiting a small stream, Þverá, in the NE‐slopes of the Hengill mountain, has influenced the genetic makeup of trout in the lake (Lagunas et al. 2023).

**FIGURE 1:**
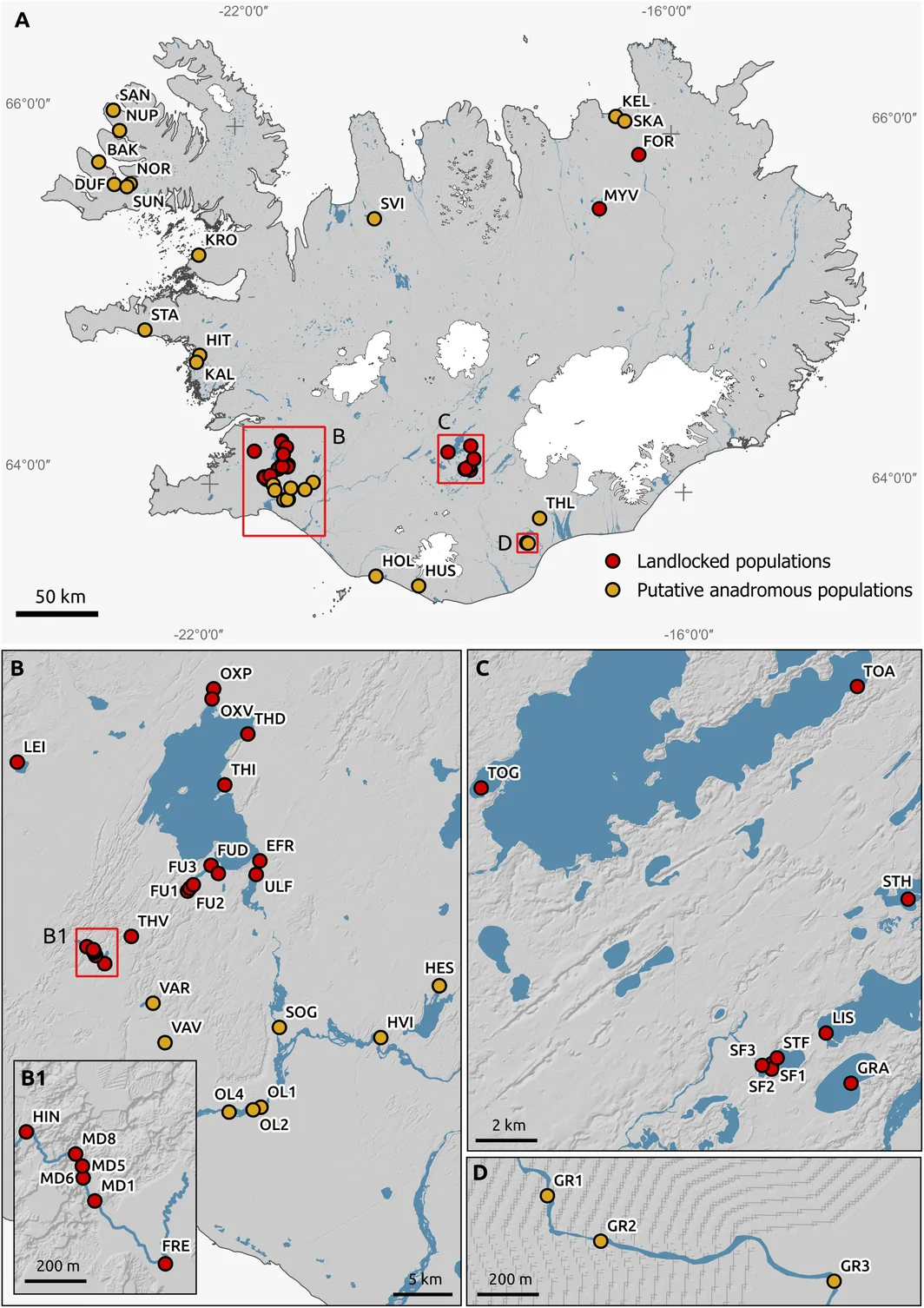
Map of Iceland showing the 58 sampling locations of this study (A). Magnified maps of two systems, that is, the watershed of River Ölfusá/Lake Þingvallavatn (B) and the Veiðivötn swarm lake system and Þórisvatn (C). Two areas are further enlarged (i.e., B1: River Hengladalsá and D: River Grenlækur). See a detailed list and coordinates in Table S1. This map was generated with the program QGIS (QGIS 2026) using data from the National Land Survey of Iceland (LMI 2020).

We sampled 1120 trout from 58 locations (13 lakes, 1 lagoon, 25 rivers) across Iceland (Figure 1, Table S1). Fourteen locations in the watershed of River Ölfusá which were part of our previous study (Lagunas et al. 2023) are indicated by a star (*) in Table S1. Large fish in lakes were sampled using nylon gillnets (25–50 mm mesh) deployed from a boat close to shore, where the depth is *ca*. 1.5 m, perpendicularly to the coastline, and left overnight. Fish were collected after 12 h, killed and either processed in situ or taken to the laboratory on ice. Spawning individuals were fished with nets (60–65 mm mesh) (River Ölfusvatnsá) or traps (River Öxará). These fish were sedated upon collection with 2‐phenoxyethanol (Pounder et al. 2018) and a small fin clip taken for DNA isolation. After recovering in a trap net the fish were returned to their habitat.

Small fish in rivers and several lakes were sampled by electrofishing using gear from KC Denmark (Silkeborg, Denmark) (Table S1). Stunned fish were retrieved with a dip net and processed at the sampling location (i.e., a small caudal fin clip was obtained). Fish were allowed to recover in a bucket with freshwater with no sedative and later returned to their habitat. Fish from the rivers Staðará and Hvítá were provided by anglers or local farmers (Table S1). As a reference group we used anadromous brown trout from Loch Slapin (Isle of Skye, Scotland).

Since we cannot always confirm that fish sampled at a certain location belong to a local spawning population, we do not use the term *population* when referring to these sampling locations. When fish were caught on spawning grounds, or the habitats were small and isolated, we assumed that these samples represented proper spawning populations (see Table S1 for sample designation).

### 2.2 DNA Isolation, Library Preparation and Sequencing

The methodology for the DNA isolation and preparation of ddRAD libraries was based on Peterson et al. (2012) and Lagunas et al. (2023). Sample DNA was digested with both *Bam*HI‐HF and *Ape*KI (see Table S2 for adapters). Thirteen libraries (1184 samples) were prepared, four of which were quality checked on an Illumina MiSeq (2 × 150 bp paired‐end). All 13 were then sequenced on an Illumina HiSeq X (2 × 150 bp paired‐end) at BGI Tech Solutions, Hong Kong. The MiSeq data was included in the analysis. For further details on library preparation see Lagunas et al. (2023).

### 2.3 Data Analysis

#### 2.3.1 Read Processing and Loci Assembling

After an initial quality check of the reads with *fastqc* (Andrews et al. 2016), we demultiplexed, removed adapter sequences, and trimmed them to 115 bp to ensure an equal read length for all samples using the script *process_radtags* from the package *Stacks* v2.4 (Catchen et al. 2011) (see Table S3 for parameters used). We followed a reference‐aligned approach running all programs within *Stacks* manually. A preliminary de novo analysis using *Stacks* v2.4 was performed to identify and select samples with coverage > 20× to ensure high quality data.

The *S. trutta* genome was downloaded from the National Centre for Biotechnology Information website (RefSeq assembly accession: GCF_901001165.1) (O'Leary et al. 2016). It was indexed with the *Burrows‐Wheeler Aligner, BWA* (Li and Durbin 2009), and the paired‐end reads aligned using the BWA‐MEM algorithm and converted to BAM files using *samtools* (Li et al. 2009), as suggested by Rochette and Catchen (2017). The programs *gstacks* and *populations*, part of *Stacks* v2.4, were run successively to align reads per sample (while calling variant sites in populations) and as a first filtering step, respectively (Table S3).

#### 2.3.2 Data Filtering

The script *paralog‐finder* v1.0 (Ortiz 2018) was used to blacklist paralogous loci based on methodology from McKinney et al. (2017). *Populations* was run again (excluding the blacklisted paralogous loci) keeping only one SNP per locus. To reduce influence from relatedness (especially in locations where juveniles were collected via electrofishing), we removed individuals with first‐degree kinship (217 total) using *vcftools* v0.1.16 (Danecek et al. 2011), applying a statistic based on Manichaikul et al. (2010) (see Table S3). Next, *vcftools* v0.1.16 was used to remove loci with a minor allele count lower than three, a minor allele frequency lower than 5%, and genotypes with less than three counts. Loci missing in more than 40% of the individuals from each sampling location were also removed. We removed loci significantly out of Hardy–Weinberg equilibrium (*p* < 0.05) in more than 1% of the populations, using the scripts from Puritz et al. (2014). After checking the distribution of missing data per individual, the inter‐quartile range (IQR) was used to calculate the exclusion threshold (i.e., Q3 + 1.5 IQR). The values of the parameters used in data filtering steps, with the resulting number of loci, are listed in Table S3. Similarly, the number of removed individuals is reported in Table S4.

We observed three individuals from two rivers in the Westfjords with a higher number of properly paired reads mapped to the *S. salar* genome than to the genome of *S. trutta* (Figure S1). One individual had already been removed during data filtering due to excessive missing data. The remaining two individuals, potentially Atlantic salmon‐brown trout hybrids, did not affect the distribution of population clusters in the PCA or phylogenetic tree analyses.

#### 2.3.3 Assessing Genetic Diversity and Relatedness

Allelic richness (*A*_R_) and principal component analysis (PCA) were calculated using the R packages *hierfstat* (Goudet 2004) and *adegenet* (Jombart and Ahmed 2011), respectively. The package radiator (Gosselin 2019) was used to determine the nucleotide diversity (Nei and Li 1979). The package *assigner* was used to calculate *F*_ST_ values (Gosselin et al. 2020). Admixture coefficients were estimated with the function *snmf* from the package *LEA* (Frichot et al. 2015), which calculates an entropy criterion that can be used to estimate the number of ancestral populations (*K*) (Frichot et al. 2014). After an initial check suggesting *K* ~ 16, we ran *snmf* 2500 times for *K* = 13–21 and chose the run and *K* value that minimized the entropy criterion. The program *RAxML‐NG* v1.2.2 (Kozlov et al. 2019) was used to calculate a maximum likelihood tree. The GTR‐GAMMA model of substitution was used, and the best scoring tree was chosen using a frequency‐based criterion and a rapid bootstrapping algorithm (Stamatakis et al. 2008).

#### 2.3.4 Testing for Association of Markers and Environmental Conditions

A larger dataset was used to screen for signals of putative adaptive divergence by retaining markers out of Hardy–Weinberg equilibrium (5614 total).

First, the function *lfmm* from the package *LEA* (Frichot et al. 2015) was used to run latent factor mixed models with temperature as the explanatory variable in a regression where the response variable was a SNP matrix (Frichot et al. 2013). Temperature was incorporated as a continuous site‐level predictor variable, using mean May–October temperatures for seven sampling locations, that is, Stóra Fossvatn, Litlisjór, Þingvallavatn, Sog, Varmá, and Hengladalsá (and streams draining therein) at two valleys (Innstidalur, Miðdalur) (Table S5). The function *snmf* was run to determine the most likely number of ancestral populations (*K*) using 100 replicates with 20,000 cycles each, with 10,000 cycles for burn‐in. The *z*‐scores of all runs were combined using the Fisher‐Stouffer method and adjusted by a genomic inflation factor which was manually reduced since it is known to be overly conservative (Frichot et al. 2015). The Benjamini‐Hochberg criterion was used to adjust for false discoveries (Benjamini and Hochberg 1995; François et al. 2016).

Second, we aimed to identify markers associating with type of habitat (i.e., lake, river) under the presumption that such markers (or linked variants) relate to the loss of anadromy or adaptation to lacustrine habitats. A two pronged approach was used: (a) to look for overall signals, all Icelandic sampling locations were classified as either lake or river, and *F*_ST_ values were calculated with the program *populations* from *Stacks* v2.4; (b) to identify local signals, we compared brown trout from each of five lakes (spawning fish from Þingvallavatn at Öxará, Stóra Fossvatn, Mývatn, Svínavatn and Hestvatn) to anadromous trout from Sog and Loch Slapin in Scotland. Pairwise *F*_ST_ values were estimated and markers with values above 0.5 were defined as putative outliers associating with lake residency. The shared and unique markers from these lakes were summarized with UpSet graphs (Lex et al. 2014) and SuperExactTests were used to test for significant overlap (Wang et al. 2015). For all approaches, the implicated SNPs were mapped onto the genome of *S. trutta* and identified using online genome browser tools (Samy et al. 2017; Sayers et al. 2022).

### 2.4 Environmental Data

Temperature data were compiled from two sources with different temporal coverage. For three sites along the Hengladalsá system (HIN, MID, FRE), temperature was recorded continuously from 15 May 2006 to 25 October 2006 (Ólafsson 2019). For all other sites, monthly measurements collected by the Marine and Freshwater Research Institute of Iceland between 2007 and 2019 were used. We calculated average temperatures for the May–October period across these years. While this approach combines single‐season and multi‐year data, the resulting thermal gradients (6.5°C–15.7°C) capture biologically meaningful variation in summer conditions relevant to trout metabolism and immune function (Scharsack and Franke 2022). It should be noted that while the single‐year data for Hengladalsá may not represent interannual variability, the temperature range across systems should be sufficient to allow detection of genomic signatures of thermal adaptation.

## 3 Results

After filtering, 2946 putative neutral markers in 600 individuals (from an initial 868) were used for diversity and relatedness analysis, while a larger dataset (5614 markers) was retained for association tests (Table S3).

### 3.1 Icelandic Brown Trout Separated Into 16 Distinct Clusters, With Headwater Groups Splitting From Their Downstream Counterparts

**FIGURE 2:**
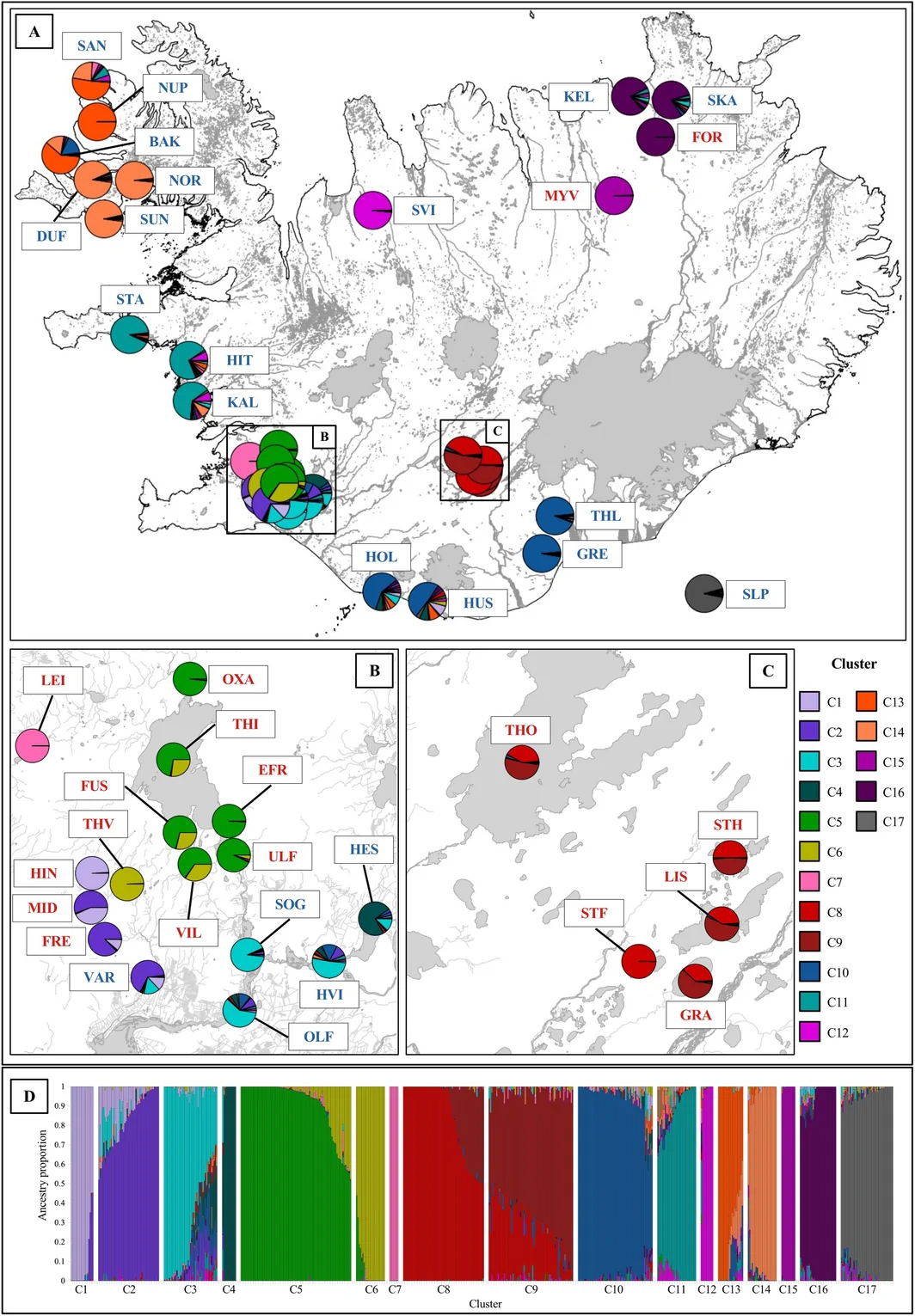
Clear genetic differentiation of brown trout in Iceland, with many distinct headwater populations and clear separation of anadromous fish by geography. (A) Map of Iceland with admixture pie plots using *K* = 17 ancestral populations. The names of headwater locations are indicated in red and those of locations with potential access to the ocean in blue. The pie for the Scottish reference group (SLP) is placed on graph for comparison. The two insets show the watersheds of River Ölfusá, Lake Þingvallavatn, Úlfljótsvatn and the Hengill mountain (B) and Veiðivötn and Þórisvatn (C). A reference to the three‐letter code for each location can be found in Table S1. (D) Ancestry composition of each fish assuming *K* = 17 grouped by cluster. Note, each cluster is formed by individuals showing > 50% of the dominant genetic component (or at the highest proportion when < 50%). A version of this matrix showing individuals grouped by location is shown in Figure S8.

**FIGURE 3:**
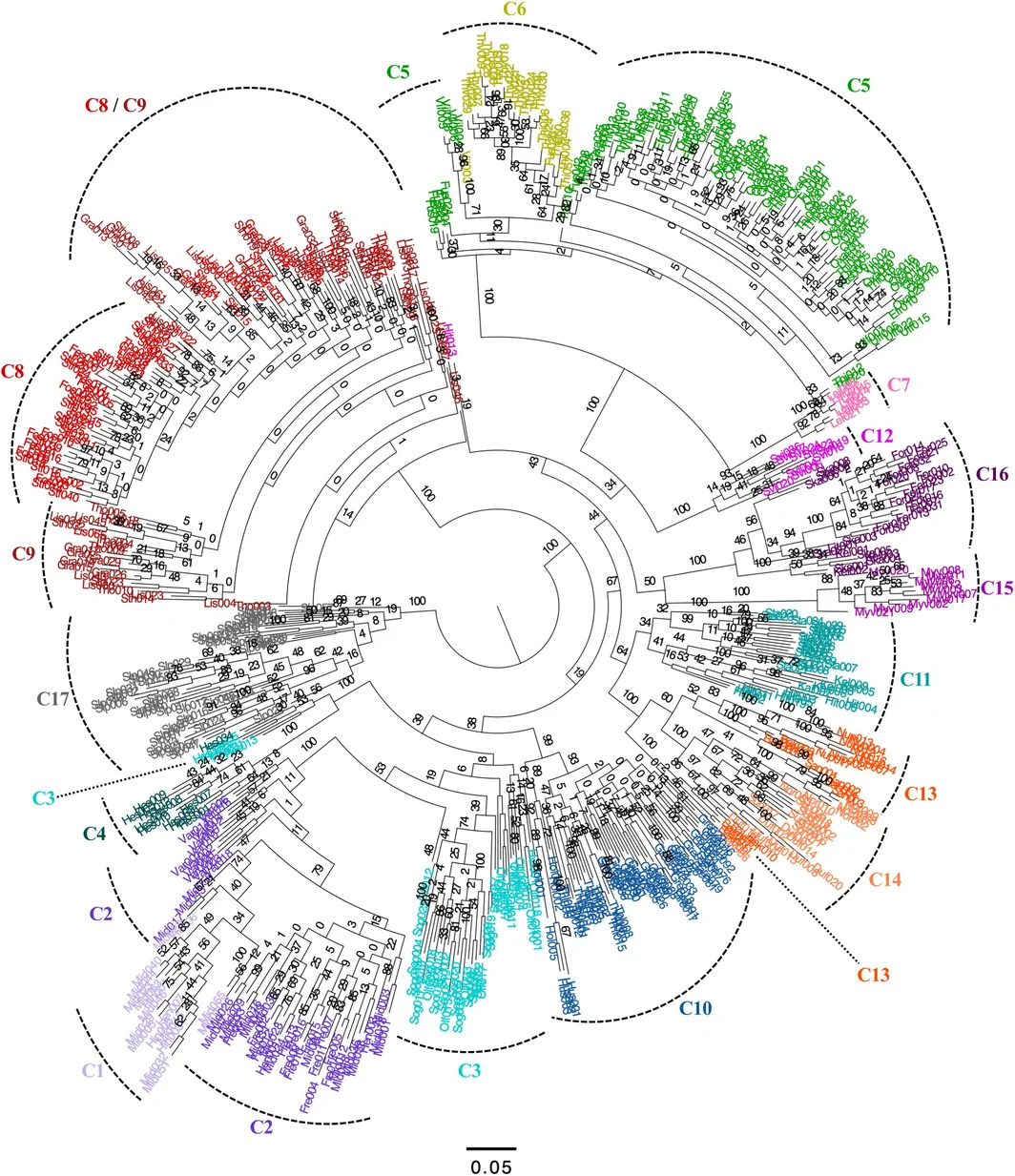
Maximum likelihood phylogenetic tree displaying 600 individuals from 40 sampling locations in Iceland, and one from the Isle of Skye, Scotland. The clusters from Figure 2 are indicated by broken lines and the colors of each fish ID as well as the cluster numbers match those from cluster C1–C17 in Figure 2. Note that not all individuals of each cluster appear on the same branch. The tree was obtained with *RAxML‐NG* using the GTR‐GAMMA model, a version of the General Time Reversible model of nucleotide substitution under the Gamma model of rate heterogeneity. After 800 replicates of bootstrapping, the topology of the tree converged to the one shown here. The numbers on major branches indicate the support values (percentages) derived from Felsenstein bootstrapping. The scale bar represents the amount of genetic change between sequences associated with the branch lengths (e.g., 0.1 represents a 10% difference between sequences per length of branch).

Headwater brown trout separated very strongly from the anadromous groups across all analyses, for example, admixture (Figure 2), phylogeny (Figure 3), PCA (Figure S2) and *F*_ST_ values (Figure S3). Admixture analyses of 41 sampling locations (including Loch Slapin, Scotland) gave a minimum entropy at *K* = 17. Although entropy values increased between *K* = 17–21 and decreased again at *K* = 22–23 (Figure S4), we favored the conservative interpretation of 17 ancestral populations (Figure 2D). Figures S5–S7, provide ancestry matrices per individual for *K* = 15–23. As well as illustrating the strong effect of isolation in headwaters, the observed clusters of sampling locations sharing a dominant ancestry component reflected the geography of rivers and lakes in Iceland (Figure 2A). Samples from the watershed of River Ölfusá formed seven distinct clusters (C1–C7), consistent with our previous findings (Lagunas et al. 2023).

All trout from the other main headwater system of Veiðivötn and Þórisvatn grouped in two clusters (C8 and C9), with a high level of mixed ancestry in all lakes except Stóra Fossvatn which had a unique ancestry where all fish grouped in cluster C8 (Figure 2C, Figure S8). All southern putative anadromous trout grouped together (C10), as did the trout in rivers flowing into the northwestern side of Faxaflói bay (C11). Fish from the northern lakes Svínavatn and Mývatn, formed unique clusters (C12 and C15, respectively). Trout from the Westfjords formed two clusters, a northern (C13) and a southern (C14) group, and the rest of the northern populations (i.e., Keldunes‐Litlaá, Skjálftavatn, Forvöð) clustered together (C16). Loch Slapin (Scotland) trout formed a unique cluster (C17). The trout inhabiting watersheds of Veiðivötn, Þingvallavatn and Úlfljótsvatn, and Leirvogsvatn were the only groups to separate from other groups on the first two principal axes of the PCA. The Scottish trout grouped with all the other Icelandic trout and only separated as a distinct group along PC4 (Figure S2). Notably, *F*_ST_ values between Loch Slapin trout and putative anadromous trout, especially those from clusters C3 (lower Ölfusá), C10 (southern), and C11 (western) (Figure S3), were much lower than *F*_ST_ values comparing the putative anadromous samples with samples from headwater localities. Trout from the headwater lakes contribute little, if any, ancestry to downstream, putatively anadromous populations. Alleles characteristic of the Þingvallavatn trout were absent from the Ölfusá tributaries, and no genetic signal from the Mývatn population was detected in the northern, putative anadromous fish.

### 3.2 Headwater Trout Sit on Well Supported Branches of a Phylogenetic Tree

The phylogenetic analysis yielded an un‐rooted tree that was largely congruent with the clusters detected in the admixture analysis assuming *K* = 17 (Figure 3). As might be expected, the two deepest well supported branches separate the Scottish trout and the trout from Iceland. The Icelandic branch split into a well‐supported Veiðivötn/Lake Þórisvatn clade, and a lower support branch (19%), containing the remaining locations. Several well supported (85%–100%) main branches stand out as well as terminal branches that represent isolated mountain stream populations. Namely, (1) a main branch with two deep branches, (a) the Þingvallavatn/Úlfljótsvatn meta‐population with seven sampling sites, OXA, THI, EFR, ULF, VIL, FUS and a well‐supported sub‐branch for the isolated population in the mountain stream Þverá (THV), and (b) the isolated population in Leirvogsvatn (LEI); (2) the populations in Svínavatn (SVI); (3) Mývatn (MYV); (4) samples from tributaries of River Jökulsá á Fjöllum (Skjálftavatn, a small recently formed lake) and a separate sub‐branch for the isolated stream at Forvöð; (5) samples from five rivers in the Westfjords, four of which branch according to site; (6) a branch representing rivers on the south coast with two sub‐branches, one with populations from Grenlækur (GRE) and Þverá á Síðu (THL) and the other with samples from two rivers on Sólheimasandur, Húsá (HUS) and Hólsá (HOL). The Veiðivötn/Þórisvatn branch generally lacked geographic structure, except for a weakly supported branch of the Stóra Fossvatn spawners (24%).

Putative anadromous individuals caught in the lower reaches of the Ölfusá/Hvítá watershed sit on many poorly supported sub‐branches except for the samples from Lake Hestvatn. The anadromous southern trout formed a very well supported single branch, that may be related to these groups (Hengladalsá/Varmá and Hestvatn/Hvítá/Sog/Ölfusá) (Figure 3).

### 3.3 Low Genetic Diversity in Most Headwater Locations

**FIGURE 4:**
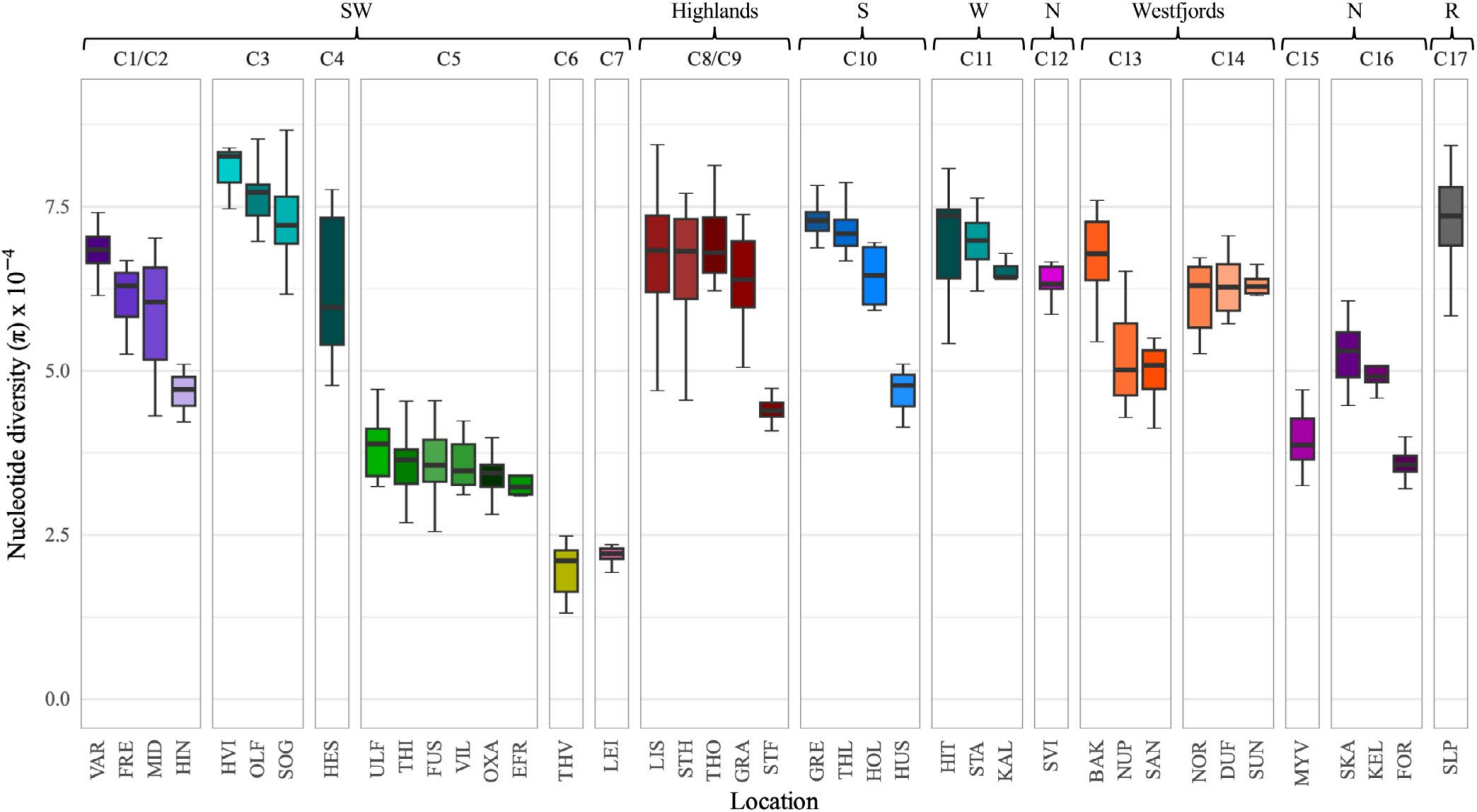
Boxplot displaying the nucleotide diversity in brown trout by location in each cluster (C1–C17). Most headwater brown trout had reduced nucleotide diversity compared to putative anadromous trout. Sites were arranged by geographic location/watershed, starting with the Varmá river in the south to two landlocked populations in the North‐east and the reference group from Loch Slapin in Scotland (gray on the far right). The three‐letter codes match those in Table S1. SW: South‐west, S: South, W: West, N: North, R: Reference group.

Most of the headwater brown trout had substantially lower genetic diversity (*π*) than the putative anadromous trout, which in turn showed similar levels to that of Loch Slapin, Scotland (Figure 4). Diversity diminished upstream, for example, Varmá up to Hengladalsá (Innstidalur) (*π* = 6.82 × 10^−4^ to 4.68 × 10^−4^) (Figure 4, Table S6). Similarly, Forvöð exhibited a low value (i.e., *π* = 3.57 × 10^−4^) in contrast to sites located downstream (i.e., Keldunes‐Litlaá with 4.98 × 10^−4^ and Skjálftavatn with 5.26 × 10^−4^) (Figure 4, Table S6). Long isolated headwaters lakes (i.e., the Þingvallavatn/Úlfljótsvatn, Mývatn, Stóra Fossvatn) also showed low diversity (i.e., *π* = 3.34–4.38 × 10^−4^) (Table S6). The lowest observed values were from populations in the mountain stream Þverá in Hengill and Leirvogsvatn, both small populations situated above impassable waterfalls (Figure 4). The putative anadromous trout in the small river Húsá in the south also had low genetic diversity (i.e., 4.673 × 10^−4^) (Figure 4, Table S6). Anadromous fish showed high allelic richness, that is, *A*_R_ = 1.31–1.87, whereas headwater locations above waterfalls and without recorded restocking (e.g., Stóra Fossvatn) showed low values *A*_R_ = 1.13–1.39 (Table S6). Lakes with known stocking (i.e., the Veiðivötn lakes, except Stóra Fossvatn) showed *A*_R_ values comparable to putatively anadromous fish, i.e., average *A*_R_ = 1.41 (Table S6).

### 3.4 Allele Frequencies at Several Loci Associate With Habitat Temperature

**FIGURE 5:**
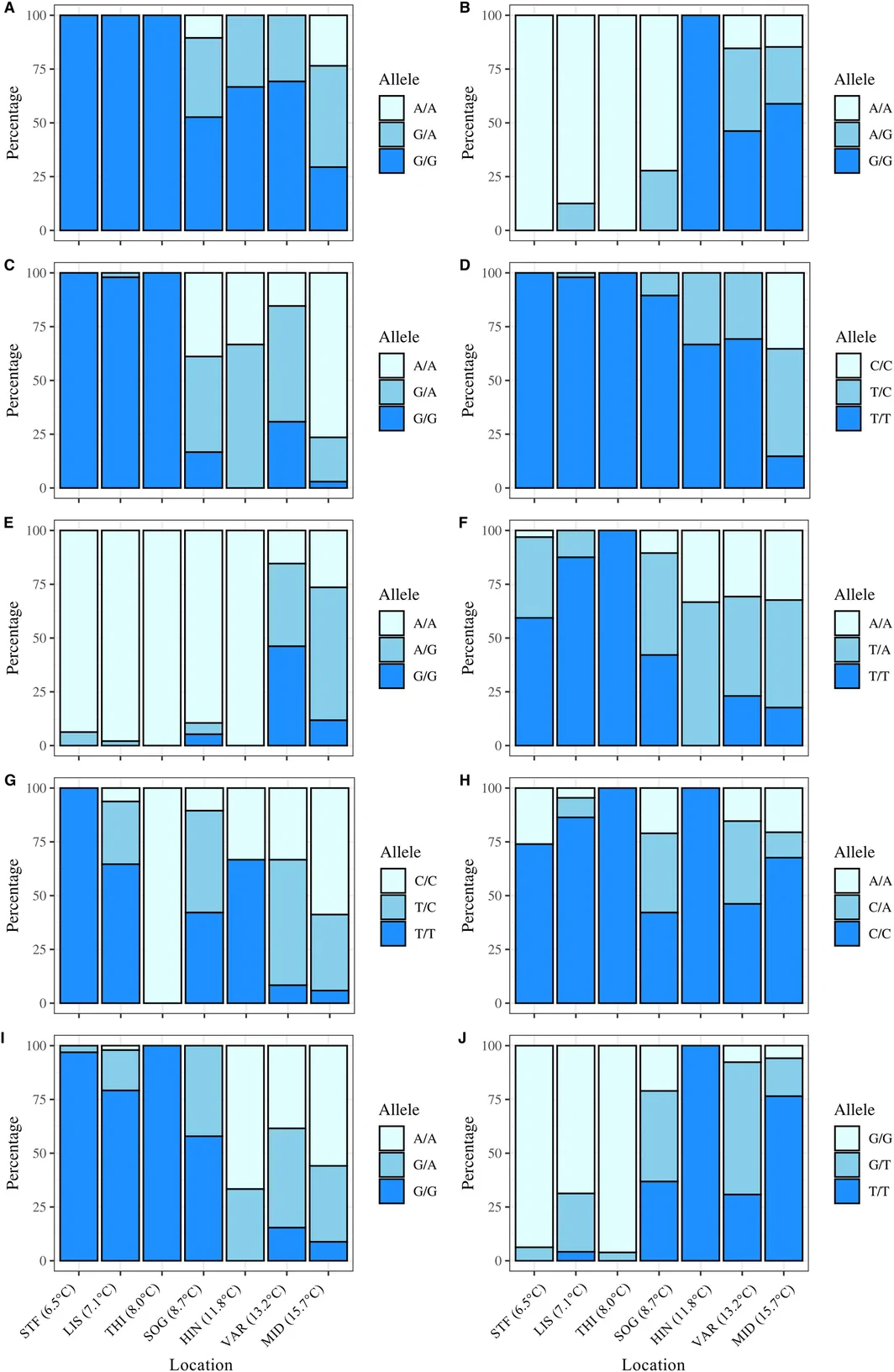
Allele frequency for 10 SNP's with the strongest association to temperature, FDR < 0.001 (Benjamini‐Hochberg algorithm). When two or more markers were present in a chromosome region, the one with the lowest *p*‐value was represented. The sampling locations are ranked by increasing average temperature in May–October (indicated on the *x*‐axis). The three‐letter codes match those in Table S1. (A) Marker in chromosome 33 (*p‐*value = 2.82 × 10^−13^) at no known gene. (B) Marker in chromosome 21 (*p*‐value = 1.29 × 10^−11^) at the coding region of a low‐density lipoprotein receptor‐related protein. (C) Marker in chromosome 15 (*p*‐value = 2.09 × 10^−11^) at no known gene. (D) Marker in chromosome 20 (*p*‐value = 2.51 × 10^−11^) at no known gene. (E) Marker in chromosome 29 (*p*‐value = 2.45 × 10^−10^) at the coding region of a unc‐13 homolog C‐like protein. (F) Marker in chromosome 9 (*p*‐value = 1.15 × 10^−9^) at the coding region of ras‐related protein Rab‐7a‐like. (G) Marker in chromosome 10 (*p*‐value = 2.19 × 10^−9^) at the coding region of an arf‐GAP with dual PH domain‐containing protein. (H) Marker in chromosome 8 (*p*‐value = 2.51 × 10^−9^) at no known gene. (I) Marker in chromosome 2 (*p*‐value = 1.70 × 10^−8^) at the coding region of a zinc finger CCHC domain‐containing protein. (J) Marker in chromosome 16 (*p*‐value = 2.04 × 10^−8^) at no known gene.

A latent factor mixed model was run with temperature as an explanatory variable, using a subset of seven sampling sites with known summer temperatures. After adjusting for multiple tests, that is, lambda = 0.45 (Figure S9) and setting *q* = 0.001 for the False Discovery Rate, 40 markers were deemed significantly related to temperature (Figure S10A), suggesting polygenic contribution to temperature adaptation. For comparison, *q* = 0.05 returned 461 markers (Figure S10B). The 40 markers mapped to 22 linkage groups, with 19 in coding regions and 21 near (< 5000 bp) coding regions (Table S7). Eleven of these markers were both on and near coding regions. Some markers were in or close to transmembrane protein genes (e.g., leucine‐rich repeat transmembrane neuronal protein 4‐like on chromosome 12). Other markers associated with lipid metabolism (e.g., long‐chain‐fatty‐acid‐CoA ligase 6‐like, transcript variant X2 in chromosome 15) and immune response (e.g., zinc finger CCHC domain‐containing protein 2‐like in chromosome 2). The pattern of allele frequency by location reflected this association with temperature (shown for 10 markers in Figure 5). In most cases, the rare allele (presumably derived) associated with increasing temperature (Figure 5). In one case, the allele common at the lowest temperature was almost completely absent at the highest temperature (Figure 5C). The allele frequency of these markers was plotted per sampling station in Figures S11 and S12.

### 3.5 Genomic Divergence Related to the Loss of Anadromy

To study the genetic differentiation associated with loss of anadromy we compared allele frequencies between lake‐ and anadromous populations. First, the overall comparison (all Icelandic locations classified as either lake or river) returned *F*_ST_ values up to 0.4 and we defined the markers with the highest 0.5% as outliers. The most extreme outliers were associated with genes with functions such as cellular energy production in mitochondria (e.g., translocase of outer mitochondrial membrane in chromosome 16) and skeletal muscle cells metabolism (e.g., dystrobrevin beta‐like transcript on chromosome 25) (Table S8 and Figure S13).

**FIGURE 6:**
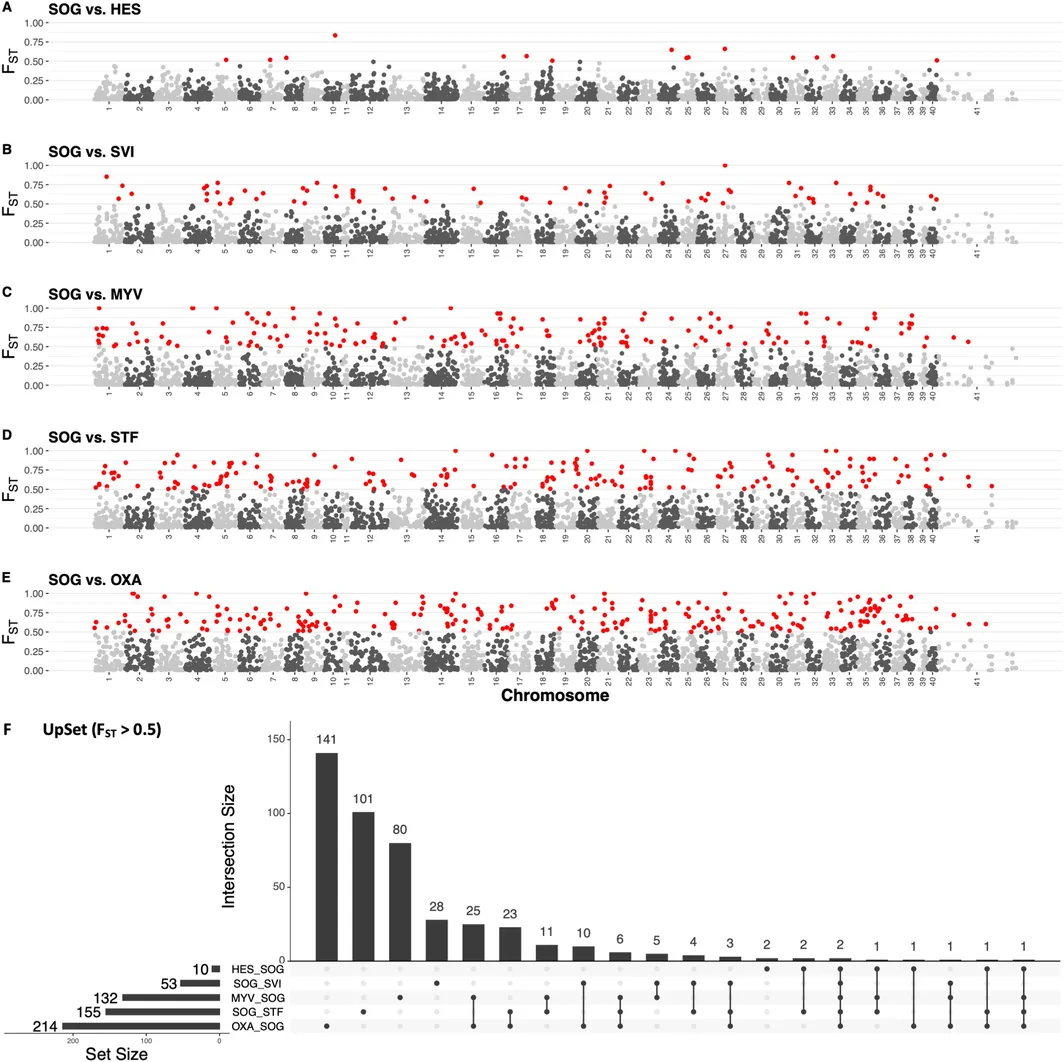
Genome wide divergence between brown trout from five lakes (i.e., Hestvatn, Svínavatn, Mývatn, Stóra Fossvatn, Þingvallavtn spawners at Öxará) and putative anadromous trout (i.e., Sog). Manhattan plots show *F*_ST_ values along the *S. trutta* genome (A–E). Markers with *F*_ST_ values > 0.5 are colored red. Linkage groups (chromosomes) are listed along the *x*‐axis and SNP coloring alternates between gray and blue to demarcate them. “Chromosome 41” is an aggregate of unplaced contigs. (F) UpSet plot displaying the overlap SNP's between population comparisons, shown as the Intersection Size, using Sog as the reference putative anadromous population. The Set Size indicates the number of significant markers for each of the comparisons. HES_SOG: Hestvatn versus Sog; SOG_SVI: Sog versus Svínavatn; MYV_SOG: Mývatn versus Sog; SOG_STF: Sog versus Stóra Fossvatn; OXA_SOG: Öxará versus Sog. See Table S1 for three‐letter codes and Figure S14 for results of exact tests on intersects.

Secondly, we used a pairwise approach, estimating *F*_ST_ values between five lakes relative to the putative anadromous trout of River Sog. We defined markers with *F*_ST_ > 0.5 as potentially associating with diverging loci (Figure 6). The outliers were located on multiple chromosomes, suggesting extensive genetic separation between these groups (Figure 6A–E). Based on the degree of separation (Figure 3), most markers separated Öxará (214), Stóra Fossvatn (155), and Mývatn (132), but fewer did for Svínavatn (53) and Hestvatn (10) (Figure 6F), two lakes that can be accessed from the sea. The majority (60%–66%) of the putative adaptive markers were unique to the three isolated headwater lakes (Figure 6F). Most of the Hestvatn markers were also found in other lakes (Figure 6F and Table S9). Only two markers were found diverging in all five lakes, 13 in three or four lakes, but most (81) of the overlapping loci were shared among two lakes. Many of these intersects were significantly larger than what may be expected by chance (Figure S14). The markers present in several comparisons were related to genes with functions such as cellular response to stimuli like osmotic and heat stress (i.e., dual‐specificity kinase in chromosome 3), growth of neuronal structures (i.e., coronin, actin binding protein in chromosome 7) or cellular mitosis (i.e., stathmin‐3‐like in chromosome 14) (Table S10). Note, only markers present in all populations were analyzed for overlap. Comparable patterns were found when the Scottish population (Loch Slapin) was used as the reference anadromous group, though the exact numbers differed (Figure S15).

## 4 Discussion

### 4.1 Patterns of Genomic Variation Within and Among Populations of Icelandic Brown Trout Reflect the Topography and History of Waterways

Genetic diversity and differentiation in freshwater fish like brown trout is heavily influenced by habitat connectivity. The stream‐hierarchy model (Meffe and Vrijenhoek 1988) predicts that fish populations in well‐connected reaches are genetically similar, whereas isolated fragments diverge because drift overwhelms gene flow (Heggenes et al. 2009). Fragmentation, whether caused by natural waterfalls or anthropogenic dams, creates smaller, more variable patches that reduce the genetic diversity of isolated populations (Fagan 2002; Neville et al. 2006), a pattern especially pronounced in island‐like or highly restricted habitats (Frankham 1998).

Our results illustrate how the patterns of genomic variation within populations and relatedness among them have been shaped in the postglacial era, on a large scale, by the geography of Icelandic watersheds, and, on a smaller scale, by geological and climatic forces molding the topology of rivers and streams within watersheds. Admixture analyses revealed 16 distinct groups following a nested hierarchy of tributaries, watersheds and areas (groups of adjacent watersheds), typical of homing salmonids. Furthermore, we observed a high degree of genetic differentiation among the isolated headwater populations (e.g., the Úlfljótsvatn‐Efra Sog‐Þingvallavatn system and Leirvogsvatn in the south‐west, as well as Mývatn in the north‐east). This strongly indicates that these populations became isolated soon after these waters were colonized at the end of the last glacial epoch and have since experienced genetic drift. Moreover, considering different ecological conditions in these headwater systems, adaptation to local environments likely played a role.

The data also show that the genetic differentiation between isolated headwater and downstream populations varies among systems. While some of the headwater populations have likely been isolated for a few thousands of generations, downstream gene flow has and may still influence lowland populations. The likelihood of downstream gene flow may hinge on the size and stability of the isolated habitats. The ancestry analyses suggest that isolated populations in small streams may have contributed alleles to downstream populations. In the south‐west, trout from Þverá (THV), a small stream on the eastern slopes of the Hengill mountain, have contributed genes to the trout in Þingvallavatn (THI), Efra‐Sog (EFR) and Úlfljótsvatn (ULF), and the small streams in Hengladalir (HIN, MID, FRE) in the western slopes of Hengill have contributed genes to trout in Varmá (VAR). The same pattern is seen in the north‐east, where trout from the small stream in Forvöð (FOR) contributed genes to downstream populations in Skjálftavatn (SKA) and Litlaá (KEL) (see Figure S8). Downstream gene flow from headwater lakes seems more restrictive or negligible. Even in a large lake such as Þingvallavatn, which presently harbors a large population of trout, we see no traces of downstream gene flow to the lowland trout. Formation of unscalable downstream barriers sets up a scenario whereby juveniles face two choices. Either migrate to the sea and attempt to mate elsewhere or stay put, mature and reproduce “at home.” If trout that got “trapped” in headwaters possessed genetic variation for traits associated with downstream migration one can envisage that individuals that migrated to the sea tended to carry the alleles associated with migration thus enhancing the evolution of residency (Zarri et al. 2022). Studies have shown that populations that encounter barriers to seaward migration may shift to residency in a few generations (Pearse et al. 2009) and, if the system includes a large lake, a pattern of freshwater migration may arise (Pearse and Campbell 2018) where trout spawn in inflowing (and/or outflowing) rivers but migrate into the lake for enhanced growth. The trout populations of Þingvallavatn in the south‐west and Mývatn in the north‐east appear to be pertinent examples of rapid evolution towards residency driven by favorable ecological conditions.

Large fish populations or meta‐populations in open river networks or lakes are likely to withstand bottleneck events that would otherwise drastically reduce genetic diversity (Neville et al. 2009). Nonetheless, fish in extensive river sections can lose diversity over geological timescales (Carim et al. 2016). The postglacial history of Icelandic freshwater systems is marked by tectonic dynamics and volcanism (Quay et al. 2019). These processes were especially prominent in the neo‐volcanic zone where extensive lava flows affected the topology of rivers, for example, by damming (Larsen 1984, 2023; Saemundsson 1992) and in some cases this may have resulted in major changes to watersheds. Apart from changing waterways, large eruptions, like the Lakagígar eruption in 1783–1984, must have had far ranging effects on freshwater ecosystems (Laufeld 1994). This eruption discharged massive amounts of hydrofluoric acid and sulfur dioxide compounds thus contaminating soils and freshwaters and causing the death of over 50% of farm animals and a famine that killed an estimated 20% of Icelanders (Karlsson 2000; Thordarson and Self 2003). Several large explosive eruptions occurred in the postglacial era some of which caused huge deposits of tephra over large areas of Iceland (Larsen 2023; Larsen and Eiríksson 2007). There can be little doubt that some of these catastrophic events must have affected freshwater ecosystems and populations of fish. To what extent the low genetic diversity observed in Þingvallavatn/Úlfljótsvatn, Stóra Fossvatn and the Mývatn populations could be bottleneck effects caused by volcanic activity is not known.

The phylogeographic history of brown trout in Western Europe has been extensively studied first employing variation at protein loci (Ferguson and Fleming 1983; Hamilton et al. 1989) and then mtDNA haplotypes (Bernatchez 2001; Cortey et al. 2009; Hynes et al. 1996; McKeown et al. 2010). The fixation of the Ldh‐5 (100) allele in Icelandic lakes supports the hypothesis of an “Ice Age trout” (i.e., an ancestral race that colonized newly formed waterways at the end of the last glacial maximum) especially when referring to the large, piscivorous trout of Þingvallavatn (Skarphéðinsson 1996). The studies based on variations in mtDNA have shown that colonization history of trout in Western Europe is complex and reflects a history of cold and warm periods throughout the Pleistocene. These fluctuations caused divergence of several clades during repeated isolations in multiple refugia followed by range expansions and mixing of clades, both before and after the last glacial epoch (Bernatchez 2001; Cortey et al. 2009; Hynes et al. 1996; McKeown et al. 2010). In a study of 81 trout populations in Ireland and Britain, McKeown et al. (2010) found 25 haplotypes, belonging to 7 two‐step clades. Most of these populations had a mixture of clades, illustrating a complex history of colonization. However, samples from several lakes in south, west, north and east of Iceland only showed a single haplotype, which likely reflects a founder effect in a single postglacial, colonizing wave followed by prolonged isolation of Iceland (McKeown et al. 2010). Considering the fluctuations in ice cover, isostatic movements and sea level during the last millennia of the last glacial epoch (~13.8 cal ky–~10 cal ky BP), a modified version of the one wave‐hypothesis seems most plausible, i.e., that colonization of trout in Iceland proceeded as one prolonged wave, where some of the early arriving fish became isolated in headwater systems, where genetic drift and/or adaptation to local conditions would reduce genetic diversity and increase differentiation among populations. The fact that the samples from Þingvallavatn and Leirvogsvatn in the south‐west, the Stóra Fossvatn population (the only lake of the Veiðivötn system not affected by stocking) in the highlands, and Mývatn in the north show relatively low genetic diversity, seems to support this scenario.

Other mechanisms causing decreased genetic diversity such as founder effects and population bottlenecks cannot be overlooked here. The colonization window of these headwater habitats may have been short, and the numbers of successful invaders only constituting a small sample of the anadromous trout colonizing Icelandic waters. Such founder effects would have reduced genetic variation from the beginning, with subsequent drift further enhancing diversity loss. Furthermore, some trout populations may have experienced severe reduction of genetic diversity caused by ecological catastrophes like volcanic eruptions.

While the lower genetic divergence between putative anadromous populations and the Scottish reference group might point towards a model of secondary contact between an ancestral form of the Scottish trout and the Icelandic putative anadromous trout, a single migratory wave prolonged in time may have the same effect as a scenario with two distinct colonization waves. A future study incorporating both anadromous and resident trout populations from the Faroe Islands, the British Isles, Norway, and Iceland, particularly those isolated by unscalable waterfalls, is needed to test these models.

The Veiðivötn system presents a good example of how both geological forces and anthropogenic intervention can shape the genetic structure of trout populations. Our data confirms that the trout population of Stóra Fossvatn exhibits the signs of long‐term isolation, a unique genetic signature (Cluster C8), that is, low nucleotide diversity, and significant genomic divergence from anadromous populations in the southern lowlands (Ölfusá/Hvítá/Sog; Grenlækur, Þverá á Síðu). In contrast, the stocked lakes, including Þórisvatn, displayed a different genetic profile than the spawners in Stóra Fossvatn. They all showed a mixture of two dominant ancestry components, that is, from the natural population in Stóra Fossvatn and a component of unknown origin (Cluster C9). This suggests that the stocking efforts have included fish from elsewhere, possibly from anadromous populations. Alternatively, the Veiðivötn system contained two separate genetic groups of brown trout and only one of them mixed when the stocking was initiated.

Connectivity and fragment size are key drivers of genetic diversity (Fagan 2002). Natural or artificial barriers that isolate brown trout populations accelerate drift and reduce diversity, while large, connected habitats with high gene flow rates mitigate these effects. Anthropogenic actions such as restocking can temporarily boost diversity in isolated systems, but they also mask natural genetic patterns and should be considered carefully in conservation planning.

### 4.2 Adaptation to Local Environmental Conditions

Adaptation to local habitats is a key element in conservation management of Salmonid populations. Large scale studies of population genetics have shown that climate variables such as summer maximum temperature and winter precipitation can explain both neutral and adaptive genetic differentiation within metapopulations (Hand et al. 2016). As climate change accelerates environmental shifts, this genomic insight may allow for the prediction of which populations possess the necessary genetic variation to withstand future climate regimes and to prioritize the protection of those unique, locally adapted lineages before they are lost.

#### 4.2.1 Allele Frequencies at Several Loci Associated With Habitat Temperature

Markers associated with temperature were found in genes encoding transmembrane proteins, parts of the immune system, and enzymes for lipid metabolism (Table S7). Temperature is known to affect the immune response of fish, through both specific mechanisms (e.g., antibody production) and nonspecific mechanisms (e.g., phagocytosis) (Scharsack and Franke 2022) and also influences pathogen growth and diversity (Morvan et al. 1998). Two of the observed genes were directly involved in defense mechanisms, that is, the zinc finger CCHC domain protein which has been found to play a role in immunity by binding to viral RNA (Lian et al. 2018), and the immunoglobulin superfamily member that encodes antibodies (Warr 1995).

The alteration of the lipid composition of the cell membranes is a well‐known response that organisms use to achieve homeoviscosity of the membranes when exposed to different temperatures (Ernst et al. 2016). In rainbow trout, *O. mykiss* this process is considered quite complex and involves specific fatty acids, whose metabolism is modified according to the affected tissue and environmental temperature (Hazel 1984). Cold temperatures are associated with a higher concentration of lipids in liver and muscle tissues compared to warm acclimated brook trout, *Salvelinus fontinalis* (Hochachka and Hayes 1962). We identified markers in genes related to transmembrane transport and lipid metabolism, that is, the genes coding for a leucine‐rich repeat transmembrane neuronal protein and a long‐chain‐fatty‐acid‐CoA ligase, which may reflect differential selection acting in different thermal regimes. However, further work is needed to confirm if these polymorphisms affect fitness and if they alter gene function or expression levels. Moreover, because temperature is spatially structured across Iceland, the observed associations may partly reflect correlated environmental or geographic variables such as altitude, latitude, or watershed history (Rellstab et al. 2015). While the latent factors in our LFMM model partially account for population structure and associated environmental confounding, the limited number of sites with temperature data (seven) restricts the power of this correction. Disentangling the effects of temperature from those of other spatially correlated gradients would benefit from: (i) genotype‐environment association analyses incorporating multiple environmental predictors, (ii) independent replication in systems where temperature varies independently of geography (e.g., geothermally heated and cold streams within the same watershed), and (iii) experimental validation through common garden experiments across thermal gradients. The biological plausibility of the candidate genes, specifically their roles in temperature‐sensitive processes such as immune function, lipid metabolism, and membrane homeostasis, supports, but does not prove, a causal role for temperature. Future studies evaluating whether these associations hold in other trout populations or in other salmonids exposed to similar thermal gradients would further strengthen the inference of temperature‐driven selection.

Additionally, the temperature data used in the LFMM analysis were compiled from two sources with different temporal coverage. For three sites along the Hengladalsá river (HIN, MID, FRE), temperature was recorded continuously from 15 May 2006 to 25 October 2006 (Ólafsson 2019). For all other sites, monthly measurements collected by the Marine and Freshwater Research Institute of Iceland between 2007 and 2019 were averaged for the May–October period. While this approach combines single‐season and multi‐year data, the resulting thermal gradients (6.5°C–15.7°C) capture biologically meaningful variation in summer conditions relevant to trout metabolism. We acknowledge that the mixed temporal sources are a limitation, but note the substantial thermal contrasts across Icelandic streams are unlikely to be artifacts due to timing of measurements, given the consistent influence of air temperature and geothermal inputs on stream temperatures. Future studies should employ stantardized simultaneous temperature monitoring across all sampling locations.

#### 4.2.2 Genomic Divergence Related to the Loss of Anadromy

By contrasting populations in five lakes against a putative anadromous trout from River Sog, we identified numerous divergent SNP‐markers, many of which are shared among lakes and may have connections with loss of anadromy and local adaptation to lake ecosystems. Several markers mapped to genes relevant in the physiology of anadromy, highlighting the genetic background of this process (Kendall et al. 2015). For example, the interphotoreceptor matrix proteoglycan is involved in the regulation of growth factors, in the development of cytoskeletons of surrounding cells, and in regulating oxygen and nutrient transport (Ishikawa et al. 2015). Coronin and stathmin‐3‐like proteins are critical for cell motility and cytoskeletal organization, which are key in the tissue remodeling required during smoltification (West et al. 2021). Dual specificity protein kinase CLK4‐like is related to osmotic and heat stress, making it relevant for the extreme salinity transitions between freshwater and seawater (Evans and Kültz 2020). Angpt2b (angiopoietin 2b) relates to vascular remodeling and endothelial cell function. Anadromous fish require robust cardiovascular adaptations for sustained swimming and osmoregulation. Reduced expression or divergence in angiopoietin pathways could indicate adaptation to the lower energetic demands of residency (Castro et al. 2013).

The high number of divergent markers (72) in Öxará could be an indication of strong selection against anadromy in Þingvallavatn trout. However, the high genetic differentiation (already observed in other analyses and mentioned above) is congruent with the hypothesis that Þingvallavatn was colonized and became isolated early in the Holocene.

Hestvatn only displayed 10 markers exceeding the *F*_ST_ > 0.5 threshold. While this may indicate some degree of differentiation associated with lake residency, the low degree of divergence as well as the high allelic richness point towards gene exchange with other populations. These results suggest that in Hestvatn there is a population of trout undergoing evolution of residency with gene flow.

The patterns of divergence revealed here argue more for unique genetic signatures associating with loss of anadromy in the resident trout populations, as rather few markers were shared among two or more of them. Our results reflect those of several other studies of genetic parallelism in salmonids (Gagnaire et al. 2013; Rennison et al. 2019; Salisbury et al. 2022), but may also mirror the limited genomic coverage of the ddRAD approach (Jacobs et al. 2020). Some degree of genetic parallelism was found in Arctic charr (Salisbury et al. 2022), three‐spine sticklebacks (Rennison et al. 2019), and whitefish (Gagnaire et al. 2013).

#### 4.2.3 Limitations of the ddRADseq Approach

A limitation of this study is the use of ddRADseq, which samples only a limited subset of the genome (Andrews et al. 2016). As a result, adaptive variation at loci outside the captured regions may not be observed, and the loci associated with temperature regimes or the evolution of residency identified here should be regarded as incomplete sets of candidates. Estimates of nucleotide diversity may also be biased, as diversity is assessed only at loci with restriction enzyme cut sites, which may not be representative of the genome as a whole. Furthermore, filtering steps designed to remove paralogous loci, while necessary for data quality, may inadvertently remove duplicated or rapidly evolving genomic regions that could harbor adaptive variants. Whole‐genome resequencing in future studies would provide a more complete picture of both neutral and adaptive variation across the Icelandic brown trout genome.

## 5 Conclusions

We describe 16 distinct genetic clusters across 40 sampling locations, revealing divergence by isolation and local adaptation. Unique headwater populations (i.e., Öxará, Þverá, upper Hengladalsá, Leirvogsvatn, Stóra Fossvatn and Forvöð), showed significantly reduced genetic diversity compared to anadromous counterparts, a pattern likely driven by adaptation and drift over millenia (Ferguson et al. 1995). Within waterways, population structure reflected waterway topology, with evidence of downstream gene flow limited to small, isolated river populations (e.g., from Þverá to Þingvallavatn). Surprisingly, downstream gene flow from large headwater lakes (i.e., Þingvallavatn and Mývatn) appears negligible, highlighting differences in connectivity based on the type of habitat. The effect of anthropogenic intervention in the Veiðivötn system was evident, on the stocked lakes displaying populations with mixed ancestry in contrast with the unique genetic signature of unstocked Stóra Fossvatn.

Beyond neutral structure, our results also reveal signals of local adaptation, with some markers associating significantly with temperature or degree of residency (lake trout vs. anadromous river trout). Whole genome sequencing and replication in more populations can further elucidate the genetics of local adaptation to thermal regimes and variation in anadromy.

Identifying unique populations via genetic analyses like in the present study is relevant for their management and can help in their conservation. Clearly, the protection of headwater populations with limited genetic diversity (e.g., Þverá, Leirvogsvatn) should be prioritized to keep them as evolutionary reservoirs and stocked lakes should be monitored for introgression signatures to avoid the loss of native gene pools.

## Author Contributions

**Marcos Lagunas:** conceptualization (equal), data curation (lead), formal analysis (lead), investigation (equal), methodology (equal), project administration (equal), writing – original draft (lead), writing – review and editing (equal). **Arnar Pálsson:** conceptualization (equal), formal analysis (equal), investigation (equal), methodology (equal), project administration (equal), supervision (equal), writing – original draft (equal), writing – review and editing (equal). **Lea Jerman‐Plesec:** formal analysis (equal), investigation (supporting), writing – original draft (supporting), writing – review and editing (supporting). **Zophonías O. Jónsson:** conceptualization (equal), formal analysis (equal), investigation (equal), methodology (equal), project administration (equal), supervision (equal), writing – original draft (equal), writing – review and editing (equal). **Sigurður S. Snorrason:** conceptualization (equal), formal analysis (equal), funding acquisition (equal), investigation (equal), methodology (equal), project administration (lead), supervision (lead), writing – original draft (equal), writing – review and editing (equal).

## Funding

This research was funded with a University of Iceland Doctoral Research Grant (project number 1535‐1533098), from a Landsvirkjun Research Fund, and an Eimskip Doctoral Grant to M.L.

## Conflicts of Interest

The authors declare no conflicts of interest.

## Supporting information


**Figure S1:** Properly paired reads mapped onto the *Salmo trutta* genome (*x*‐axis) versus properly paired reads mapped onto the *Salmo salar* genome (*y*‐axis). Three individuals stand out as showing a higher number of paired reads onto the *S. salar* genome. Bak012 (8.58% more reads on *S. salar* ) was removed during the data filtering steps due to a high degree of missing data. Bak006 and Duf020 4.2.1.(0.66% and 1.17% more reads on *S. salar* , respectively) were retained in the dataset and did not seem to affect the data analyses.
**Figure S2:** Principal component analysis plot showing individuals along the first six components that explained 33% of the data variation. Two sets of headwater trout separated very strongly but the Scottish reference group only separated in PC4. Panel A: PC1 = 12% and PC2 = 8.8%. Panel B: PC3 = 4.5% and PC4 = 3.1%. Panel C: PC5 = 2.5% and PC6 = 2.1%. Locations were grouped by geographic area and color coded accordingly. Northern sites are shown in magenta hues, Westfjords locations in orange tones, Western ones in teal, Southern in dark blue, Ölfusá‐Hvítá watershed locations in light blue and dark blue, and trout from the SW‐slopes of the Hengill mountain Varmá in purple hues (Hengladalsá). Þingvallavatn watershed locations in green tones, Veiðivötn and Þórisvatn in red, Leirvogsvatn in pink, and the Scottish reference group in gray.
**Figure S3:** Average pairwise *F*_ST_ scores for all locations plotted in a heatmap. Sites were grouped by geographic location/watershed, starting with the Varmá river in the south to two landlocked populations in the north‐east and the reference group from Loch Slapin in Scotland. A reference to the three‐letter code for each location can be found in Table S1. The colors of each location match those of Figure 4 and Figure S5. *F*_ST_ was estimated from Weir and Cockerham's formula using the R package *assigner* (Gosselin et al. 2020).
**Figure S4:** Cross‐entropy criterion for ancestral population estimation values (*K*) between 15 and 23 generated with the *R* package *LEA* on 2946 markers from 600 Brown trout individuals from 58 sampling locations.
**Figure S5:** Ancestry matrix generated with *K* = 15–17. Note that not all the samples from each location are grouped in the same clusters. At *K* = 16, Svínavatn collapses into a group containing western locations and at *K* = 15 the two Westfjords clusters merge into one.
**Figure S6:** Ancestry matrix generated with *K* = 18–20. Note that not all the samples from each location are grouped in the same clusters. From *K* = 20 to *K* = 19 two subgroups from the Scottish reference group Slapin collapse into one and samples from Skjálftavatn join the bigger cluster of Forvöð individuals. From *K* = 19 to *K* = 18 a third cluster from the Veiðivötn system disappears as well as a sub cluster containing samples from Kálfalækur.
**Figure S7:** Ancestry matrix generated with *K* = 21–23. Note that not all the samples from each location are grouped in the same cluster. Moving from *K* = 23 to *K* = 22 a third cluster from the Scottish reference group Slapin disappears. From *K* = 22 to *K* = 21 a subgroup of Westfjords samples, mostly from Bakkadalsá, joins another Westfjords cluster. A sub cluster of Ölfusá samples is absorbed in the Sog cluster, and the Loch Slapin cluster is subdivided in two groups.
**Figure S8:** Ancestry composition of each fish when *K* = 17 grouped by location.
**Figure S9:** Histogram of test significance values (*p*‐values) after adjusting with a genomic inflation factor (lambda parameter) = 0.45. This parameter is the median of squared *z*‐scores divided by the median of the *χ* ^2^ distribution. Neutral loci are expected to show a uniform distribution and loci under selection are expected to display a peak close to zero. Ensuring this (i.e., that the null hypothesis *H*_O_ of selective neutrality is correct) is a key requisite before applying a false discovery rate algorithm (François et al. 2016).
**Figure S10:** Manhattan plot along the *S. trutta* genome for markers tested for association with temperature in eight sampling sites in Iceland (i.e., Stóra Fossvatn, Litlisjór, Þingvallavatn, Sog, Varmá, and in the Hengladalsá river at the Innstidalur, Miðdalur, and Fremstidalur valleys). The markers associated significantly with temperature are shown in red, below the FDR = 0.001 (Panel A) and FDR = 0.05 (Panel B) (negative log of the *p*‐values is shown on the *y*‐axis). Markers are depicted along linkage groups (chromosomes) along the *x*‐axis and SNP coloring of non‐associating markers alternates between gray and blue to demarcate linkage groups. Note “Chromosome 41” is an aggregate of unplaced contigs.
**Figure S11:** Allele frequency for five SNP's with the strongest association to temperature in selected chromosomes, FDR < 0.001 (Benjamini‐Hochberg algorithm), in all locations (association was only tested for eight locations with environmental data). The three‐letter codes match those in Table S1. A: marker in chromosome 33 (*p*‐value = 2.82 × 10^−13^) at no known gene. B: marker in chromosome 21 (*p*‐value = 1.29 × 10^−11^) at the coding region of a low‐density lipoprotein receptor‐related protein. C: marker in chromosome 15 (*p*‐value = 2.09 × 10^−11^) at no known gene. D: marker in chromosome 20 (*p*‐value = 2.51 × 10^−11^) at no known gene. E: marker in chromosome 29 (*p*‐value = 2.45 × 10^−10^) at the coding region of a unc‐13 homolog C‐like protein.
**Figure S12:** Allele frequency for five SNP's with the strongest association to temperature in selected chromosomes, FDR < 0.001 (Benjamini–Hochberg algorithm), in all locations. The three‐letter codes match those in Table S1. F: marker in chromosome 9 (*p*‐value = 1.15 × 10^−9^) at the coding region of ras‐related protein Rab‐7a‐like. G: marker in chromosome 10 (*p*‐value = 2.19 × 10^−9^) at the coding region of an arf‐GAP with dual PH domain‐containing protein. H: marker in chromosome 8 (*p*‐value = 2.51 × 10^−9^) at no known gene. I: marker in chromosome 2 (*p*‐value = 1.70 × 10^−8^) at the coding region of a zinc finger CCHC domain‐containing protein. J: marker in chromosome 16 (*p*‐value = 2.04 × 10^−8^) at no known gene.
**Figure S13:** Manhattan plot along the *S. trutta* genome showing *F*_ST_ values along the genome comparing populations from two contrasting habitat types (i.e., lake vs. river). Markers that are responsible for the highest 0.5% of the *F*_ST_ values are shown in red. Linkage groups (chromosomes) are listed along the *x*‐axis and SNP coloring alternates between gray and blue to demarcate them. Note “Chromosome 41” is an aggregate of unplaced contigs.
**Figure S14:** Tests of signficance of overlap of markers found to be associated with differences in habitat in two or more lakes, using the SuperExactTest (Wang et al. 2015). On top of each bar are the numbers of genes found in each union, and the total number of genes in each comparison on the right side. Note that this is not identical to UpSet graphs, as the counts of genes in overlapping two rivers also include genes found in unions of those two rivers and others. Therefore the heigth of the columns differs between this graph and Figure 6.
**Figure S15:** Genome wide divergence between brown trout from five lakes (i.e., Hestvatn, Svínavatn, Mývatn, Stóra Fossvatn, Þingvallavtn spawners at Öxará) and putative anadromous trout (i.e., Loch Slapin, Scotland) shown in Manhattan plots of *F*_ST_ values along the *S. trutta* genome (Panels A–E). This represents the same type of analyses as in Figure 6, but in this case Scotland trout was used as reference point instead of river Sog. Markers with *F*_ST_ values > 0.5 are highlighted in red. Linkage groups (chromosomes) are listed along the *x*‐axis and SNP coloring alternates between gray and blue to demarcate them. Note “Chromosome 41” is an aggregate of unplaced contigs. Panel F: UpSet plot displaying the overlap SNP's between population comparisons, shown as the Intersection Size, using Loch Slapin as the reference putative anadromous population. The set size indicates the number of significant markers for each of the comparisons. HES_SCO: Hestvatn versus Loch Slapin; SCO_SVI: Loch Slapin versus Svínavatn; MYV_SCO: Mývatn versus Loch Slapin; SCO_STF: Loch Slapin versus Stóra Fossvatn; OXA_SCO: Öxará versus Loch Slapin. The three‐letter codes match those in Table S1.

**Table S1:** Location of 59 sampling sites in 41 water bodies grouped by geographical area/watershed. The three‐letter code for each location used throughout this study is indicated in the second column. The collection methods were electro‐fishing (E), net fishing (N), trapping (T), and angling (A). Fish were defined for each sample (D): S: spawners; L: local juveniles and adults; U: individuals from unidentified population. The number of samples sequenced per location (*n*) and sampling year are also shown. A star * indicates sampling stations included in a previous study on the genetic structure of brown trout in the Þingvallavatn/Ölfusá watersheds (Lagunas et al., 2023). When multiple stations were present in a water body, the full ID of the station is also shown (as it appears in Figure 1). Note that sampling locations were combined during analyses and thus the three‐letter ID code was unified for many of them.
**Table S2:** List of barcodes for each adapter used in the preparation of the ddRADseq libraries. Eight *Bam*HI‐HF adapters were coupled with 12 *Ape*KI adapters to generate 96 unique combinations.
**Table S3:** List of relevant parameters for each program used at every stage of the data processing and filtering. The number of resulting loci at the end of each step is also shown.
**Table S4:** Number of individuals that were filtered out at different stages of data processing. N_SEQ_: number of samples sequenced per location; N_ALG_: samples that were aligned to the *Salmo trutta* genome after a preliminary quality control (i.e., samples with coverage < 20X when using a de novo approach in Stacks were discarded); N_KIN_: individuals removed due to a high relatedness score; N_MIS_: individuals missing more than Q3 + (1.5 IQR) threshold. The three‐letter codes follow Table S1.
**Table S5:** Mean water temperature (May–October average), standard deviation, number of years sampled, and source for each of the seven sites used in the LFMM analysis. MFRI: Marine and Freshwater Research Institute of Iceland.
**Table S6:** Genetic diversity parameters for each of the 40 locations sampled, indicated by their population code and grouped by geographic location. Code: the three‐letter codes follow Table S1; *N*: sample size; *H*_O_: observed heterozygosity; *H*_E_: expected heterozygosity; AR: allelic richness; Π: nucleotide diversity. Underlined *H*_O_ and *H*_E_ values indicate significantly different values (*p* < 0.05) and the resulting effect is indicated in the last column.
**Table S7:** Markers significantly associating with temperature (FDR < 0.001) in eight Icelandic populations of brown trout. The genomic position of SNPs, *p*‐value, gene ID and product (if in a coding region) are given for each of the 40 SNPs. The ID and corresponding product of genes located within 5000 bp are also shown. Selected markers with high *p*‐values (indicated with an asterisk) were used to plot allele frequencies in Figure 5. When more than one marker was present in the same chromosome, the marker with the highest *p*‐value was chosen.
**Table S8:** Markers with F_ST_ values in the top 0.5% of the distribution along the *Salmo trutta* genome, reflecting the differences between selected populations of two contrasting habitats (i.e., lake vs. river). The genomic position of SNPs, F_ST_ value, gene ID and product (if in a coding region) are given for each of the 22 SNPs. The ID and corresponding product of genes located within 5000 bp are also shown.
**Table S9:** Overlapping SNPs between population comparisons, shown as the Intersection Size, using Sog as the reference putative anadromous population with special emphasis on Hestvatn.
**Table S10:** Markers present in most of the comparisons between five lakes (i.e., spawning fish from Þingvallavatn at Öxará, Stóra Fossvatn, Mývatn, Svínavatn, and Hestvatn) to anadromous brown trout from Sog, shown in Figure 6. The genomic position of SNPs, gene ID and product (if in a coding region) are given for each of the 15 SNPs. The ID and corresponding product of genes located within 5000 bp are also shown.

## Data Availability

The raw sequences used in this study are available at the NCBI Sequence Read Archive with accession number PRJNA996765. The link to the corresponding BioProject is: https://www.ncbi.nlm.nih.gov/bioproject/PRJNA996765/. Additionally, the code (R and other software) used during analysis is available on both GitHub and a Dryad repository:https://github.com/salmo‐trutta/ddRADseq‐data‐analysis‐Icelandic‐brown‐trout; https://doi.org/10.5061/dryad.6t1g1jxf4.
