# Supplementary material for: Genetic Structure and Signs of Local Adaptation in Icelandic Brown Trout Populations
Source: Ecol Evol. 2026 Sep 11;16(9):e74274. doi: 10.1002/ece3.74274 (PMC13564247; doi:10.1002/ece3.74274)
Supplement: Supplementary file 1 [file ECE3-16-e74274-s001.docx]

**Table S1**: Location of 59 sampling sites in 41 water bodies grouped by geographical area/watershed. The three-letter code for each location used throughout this study is indicated in the second column. The collection methods were electro-fishing (E), net fishing (N), trapping (T), and angling (A). Fish were defined for each sample (D): S: spawners; L: local juveniles and adults; U: individuals from unidentified population. The number of samples sequenced per location (n) and sampling year are also shown. A star * indicates sampling stations included in a previous study on the genetic structure of brown trout in the Þingvallavatn/Ölfusá watersheds (Lagunas *et al.*, 2023). When multiple stations were present in a water body, the full ID of the station is also shown (as it appears in Figure 1). Note that sampling locations were combined during analyses and thus the three-letter ID code was unified for many of them.

| **Name** | **Code** | **Habitat** | **Coordinates** | **Method** | **D** | **n** | **Year** |
| --- | --- | --- | --- | --- | --- | --- | --- |
| **Putative anadromous populations** | | | | | | | |
| *Northern* | | | | | | | |
| Keldunes - Litlaá | KEL | river | 66º 06.413 N; 16º 45.211 W | N/A | U | 4 | 2016 |
| Skjálftavatn | SKA | lake | 66º 04.766 N; 16º 37.936 W | N/A | U | 8 | 2013 |
| Svínavatn | SVI | lake | 65º 31.494 N; 20º 02.233 W | N | U | 36 | 2015 |
| *Westfjords* | | | | | | | |
| Bakkadalsá | BAK | river | 65º 44.160 N; 23º 48.718 W | E | U | 12 | 2016 |
| Dufansdalsá | DUF | river | 65º 37.211 N; 23º 34.342 W | E | U | 22 | 2016 |
| Norðdalsá | NOR | river | 65º 37.893 N; 23º 21.409 W | E | U | 10 | 2016 |
| Núpsá | NUP | river | 65º 55.366 N; 23º 34.314 W | E | U | 19 | 2018 |
| Sandsá | SAN | river | 66º 01.963 N; 23º 41.074 W | E | U | 4 | 2018 |
| Sunndalsá | SUN | river | 65º 35.858 N; 23º 23.945 W | E | U | 19 | 2016 |
| *Western* | | | | | | | |
| Hítará | HIT | river | 64º 42.625 N; 22º 14.867 W | E | U | 22 | 2018 |
| Kalfalækur | KAL | river | 64º 40.232 N; 22º 16.989 W | E | U | 9 | 2017 |
| Krossá | KRO | river | 65º 16.044 N; 22º 21.584 W | E | L | 36 | 2017 |
| Staðará | STA | river | 64º 49.611 N; 22º 59.242 W | A, E | L | 31 | 2017 |
| *Southern* | | | | | | | |
| Grenlækur (site 1, GR1) | GRE | river | 63º 44.102 N; 17º 59.176 W | E | U | 45 | 2017 |
| Grenlækur (site 2, GR2) | GRE | river | 63º 44.021 N; 17º 58.961 W | E | U | 23 | 2017 |
| Grenlækur (site 3, GR3) | GRE | river | 63º 43.949 N; 17º 58.022 W | E | U | 24 | 2017 |
| Þverá á Síðu (lindir) | THL | river | 63º 52.265 N; 17º 49.381 W | E | U | 18 | 2015 |
| Holtsós | HOL | tidal lagoon | 63º 32.169 N; 19º 51.600 W | N | U | 5 | 2018 |
| Húsá | HUS | river | 63º 29.324 N; 19º 19.565 W | E | U | 4 | 2018 |
| *Ölfusá watershed* | | | | | | | |
| Hestvatn* | HES | lake | 64º 02.578 N; 20º 41.682 W | N | L | 38 | 2015, 2017 |
| Hvítá* | HVI | river | 64º 00.044 N; 20º 47.617 W | A | U | 7 | 2015, 2017 |
| Ölfusá (site 1, OL1)* | OLF | river | 63º 57.058 N; 20º 59.173 W | E | U | 3 | 2017 |
| Ölfusá (site 2, OL2)* | OLF | river | 63º 56.536 N; 20º 59.880 W | E | U | 7 | 2017 |
| Ölfusá (site 4, OL4)* | OLF | river | 63º 56.396 N; 21º 00.688 W | E | U | 8 | 2017 |
| Sog* | SOG | river | 64º 00.280 N; 20º 58.375 W | E | L | 24 | 2015 |
| Varmá (Reykjakot, VAR)* | VAR | river | 64º 01.085 N; 21º 11.859 W | E | L | 7 | 2015 |
| Varmá (Vellir, VAV)* | VAR | river | 63º 59.288 N; 21º 10.363 W | E | L | 11 | 2015 |
|  |  |  |  |  |  |  |  |
| **Head-water populations** | | | | | | | |
| *Northern* | | | | | | | |
| Mývatn | MYV | lake | 65º 35.537 N; 17º 00.172 W | N | U | 31 | 2015 |
| Forvöð | FOR | river | 65º 53.414 N; 16º 27.038 W | E | U | 33 | 2016 |
| *Þingvallavatn watershed* | | | | | | | |
| Efra Sog* | EFR | river | 64º 07.955 N; 21º 01.364 W | E | L | 15 | 2015, 2017 |
| Ölfusvatnsá (site D, FUD)* | FUS | river | 64º 07.625 N; 21º 06.524 W | E | L | 12 | 2015 |
| Ölfusvatnsá (site 1, FU1)* | FUS | river | 64º 06.373 N; 21º 08.863 W | N | S | 16 | 2017 |
| Ölfusvatnsá (site 2, FU2)* | FUS | river | 64º 06.506 N; 21º 08.651 W | N | S | 11 | 2017 |
| Ölfusvatnsá (site 3, FU3)* | FUS | river | 64º 06.682 N; 21º 08.291 W | N | S | 5 | 2017 |
| Öxará (Prestakrókur, OXP)* | OXA | river | 64º 15.551 N; 21º 07.155 W | T | S | 19 | 2015 |
| Öxará (Valhallarbrú, OXV)* | OXA | river | 64º 15.350 N; 21º 07.379 W | T | S | 12 | 2015 |
| Úlfljótsvatn* | ULF | lake | 64º 07.315 N; 21º 01.677 W | N | U | 32 | 2015, 2017 |
| Villingavatnsá* | VIL | river | 64º 07.267 N; 21º 05.701 W | E | L | 11 | 2015, 2017 |
| Þingvallavatn (Ólafsdráttur, THD)* | THI | lake | 64º 13.811 N; 21º 03.362 W | N | U | 13 | 2017 |
| Þingvallavatn (Svínanes, THI)* | THI | lake | 64º 11.394 N; 21º 05.526 W | N | U | 47 | 2015, 2016, 2017 |
| *Hengill mountain* | | | | | | | |
| Þverá/upper Ölfusvatnsá* | THV | river | 64º 04.127 N; 21º 14.552 W | E | L | 36 | 2016 |
| Hengladalsá – Innstidalur* | HIN | river | 64º 03.530 N; 21º 19.197 W | E | L | 6 | 2015 |
| Hengladalsá – Miðdalur (stream 14, M14)* | MID | river | 64º 03.460 N; 21º 18.733 W | E | L | 2 | 2015 |
| Hengladalsá – Miðdalur (stream 8, MD8)* | MID | river | 64º 03.416 N; 21º 18.511 W | E | L | 18 | 2015 |
| Hengladalsá – Miðdalur (stream 6, MD6)* | MID | river | 64º 03.346 N; 21º 18.410 W | E | L | 5 | 2015 |
| Hengladalsá – Miðdalur (stream 5, MD5)* | MID | river | 64º 03.276 N; 21º 18.390 W | E | L | 18 | 2015 |
| Hengladalsá – Miðdalur (stream 1, MD1)* | MID | river | 64º 03.143 N; 21º 18.214 W | E | L | 27 | 2015 |
| Hengladalsá – Fremstidalur (stream 12, FRE)* | FRE | river | 64º 02.794 N; 21º 17.207 W | E | L | 16 | 2015 |
| *Veiðivötn system* | | | | | | | |
| Grænavatn* | GRA | lake | 64º 08.237 N; 18º 42.135 W | N | U | 32 | 2017 |
| Litlisjór* | LIS | lake | 64º 09.248 N; 18º 43.320 W | E, N | U | 67 | 2017 |
| Litla Fossvatn (site 1, SF1) | STF | lake | 64º 08.614 N; 18º 45.815 W | E | L | 9 | 2017 |
| Litla Fossvatn (site 2, SF2) | STF | lake | 64º 08.503 N; 18º 45.818 W | E | L | 5 | 2017 |
| Litla Fossvatn (site 3, SF3) | STF | lake | 64º 08.574 N; 18º 46.262 W | E | L | 5 | 2017 |
| Stóra Fossvatn | STF | lake | 64º 08.730 N; 18º 45.157 W | N | U | 41 | 2017 |
| Stóra Hraunvatn* | STH | lake | 64º 11.980 N; 18º 39.584 W | N | U | 36 | 2017 |
| *Other* | | | | | | | |
| Leirvogsvatn* | LEI | lake | 64º 11.896 N; 21º 27.742 W | N | L | 65 | 2015 |
| Þórisvatn (Austurbotn, TOA) | THO | lake | 64º 16.277 N; 18º 42.066 W | N | U | 13 | 2017 |
| Þórisvatn (Grasatangi, TOG) | THO | lake | 64º 14.108 N; 18º 59.519 W | N | U | 6 | 2017 |
| ***Reference group*** | | | | | | | |
| Loch Slapin (Isle of Skye, Scotland) | SLP | marine loch | 57º 10.491 N; 06º 01.395 W | N | U | 64 | 2017 |
| TOTAL | | | | | | 1184 |  |

**Table S2**: List of barcodes for each adapter used in the preparation of the ddRADseq libraries. Eight *Bam*HI-HF adapters were coupled with 12 *Ape*KI adapters to generate 96 unique combinations.

| **Restriction enzyme** | **Adapter name** | **Barcode** |
| --- | --- | --- |
| *Bam*HI-HF | Bam 04 | TAGAGC |
|  | Bam 05 | TATCTC |
|  | Bam 06 | AGGCAT |
|  | Bam 07 | TCCACT |
|  | Bam 08 | TTAGGT |
|  | Bam 09 | ACATAT |
|  | Bam 10 | TACGAT |
|  | Bam 11 | ACTGGC |
| *Ape*KI | Ape 02 | TTCTC |
|  | Ape 03 | TCGTT |
|  | Ape 09 | CTGTA |
|  | Ape 11 | ACCGT |
|  | Ape 13 | GCTTA |
|  | Ape 15 | GGTGT |
|  | Ape 17 | AGGAT |
|  | Ape 19 | ATTGA |
|  | Ape 21 | CATCT |
|  | Ape 23 | CCTAC |
|  | Ape 25 | GAGGA |
|  | Ape 27 | GGAAC |

**Table S3**: List of relevant parameters for each program used at every stage of the data processing and filtering. The number of resulting loci at the end of each step is also shown.

| **Stage** | **Program/script** | **Parameter** | **Description** | **Value** | **Number of loci at end of stage** |
| --- | --- | --- | --- | --- | --- |
| Assembly of loci | process_radtags (Stacks) | -D | capture discarded reads to a file | N/A |  |
|  |  | -r | rescue barcodes and RAD-Tags | N/A |  |
|  |  | -t | truncate final read length to value (bp) | 115 |  |
|  |  | --renz_1 | restriction enzyme #1 | apeKI |  |
|  |  | --renz_2 | restriction enzyme #2 | bamHI |  |
|  |  | --inline_inline | barcode is inline with sequence | N/A |  |
|  |  | -i | input file type | gzfastq |  |
|  |  | -E | encoding of quality scores | phred33 |  |
|  |  | --clean | remove any read with an uncalled base | N/A |  |
|  |  | --adapter_1 | adapter sequence #1 | AGATCGGAAGAGCGTCGTGTAGGGAAAGAGTGT |  |
|  |  | --adapter_2 | adapter sequence #2 | AGATCGGAAGAGCGGTTCAGCAGGAATGCCGAG |  |
|  |  | --adapter_mm | mismatches allowed in the adapter sequence | 2 |  |
|  |  | --paired | files are paired | N/A |  |
|  |  | --quality | discard reads with low quality scores | N/A |  |
|  | populations (Stacks) | -r | min. % of individuals in a pop. required to process a locus | 0.66 |  |
|  |  | --min_maf | min. minor allele freq. | 0.05 |  |
|  |  | --max_obs_het | max. observed heterozygosity | 0.6 | 79,274 |
| Removing paralogous sequences | paralog-finder | --minD | min. read ratio deviation | -4 |  |
|  |  | --maxD | max. read ratio deviation | +4 |  |
|  |  | --maxH | max. proportion of heterozygotes in a locus | 0.4 |  |
|  | populations (Stacks) | --write-single-snp | restrict data to only the first SNP per locus | N/A |  |
|  |  | -r | min. % of individuals in a pop. required to process a locus | 0.66 |  |
|  |  | --blacklist | loci blacklisted by paralog-finder were input to populations | N/A |  |
|  |  | --min_maf | min. minor allele frequency | 0.05 |  |
|  |  | --max_obs_het | max. observed heterozygosity | 0.6 | 38,127 |
| Data filtering | vcftools | --relatedness2 | calculation of statistic used to determine degree of kinship | N/A |  |
|  |  | --mac | min. minor allele count | 3 |  |
|  |  | --maf | min. minor allele freq. | 0.05 |  |
|  |  | --minDP | min. genotype freq. | 3 | 24,661 |
| Removing missing loci | vcftools | --exclude-positions | loci missing in > 40% of each population were removed | N/A | 5,614 |
| Removing loci out of HWE | filter_hwe_by_pop.pl | -h | min. cutoff for HWE p-value | 0.05 |  |
|  |  | -c | allowed % of all pop. that a locus can be below cutoff | 0.01 | 2,946 |

**Table S4**: Number of individuals that were filtered out at different stages of data processing. N_SEQ_: number of samples sequenced per location; N_ALG_: samples that were aligned to the *Salmo trutta* genome after a preliminary quality control (*i.e.,* samples with coverage < 20X when using a *de novo* approach in Stacks were discarded); N_KIN_: individuals removed due to a high relatedness score; N_MIS_: individuals missing more than Q3 + (1.5 IQR) threshold. The three-letter codes follow Table S1.

| **Name** | **Code** | **N_SEQ_** | **N_ALG_** | **N_KIN_** | **N_MIS_** |  |
| --- | --- | --- | --- | --- | --- | --- |
| **Putative anadromous populations** | | | | | | |
| *Northern* | | | | | | |
| Keldunes - Litlaá | KEL | 4 | 4 |  |  |  |
| Skjálftavatn | SKA | 8 | 8 |  |  |  |
| Svínavatn | SVI | 36 | 13 |  | -4 |  |
| *Westfjords* | | | | | | |
| Bakkadalsá | BAK | 12 | 12 | -2 | -1 |  |
| Dufansdalsá | DUF | 22 | 22 | -14 |  |  |
| Norðdalsá | NOR | 10 | 9 | -3 |  |  |
| Núpsá | NUP | 19 | 17 | -9 |  |  |
| Sandsá | SAN | 4 | 4 |  |  |  |
| Sunndalsá | SUN | 19 | 18 | -10 |  |  |
| *Western* | | | | | | |
| Hítará | HIT | 22 | 22 | -13 |  |  |
| Kalfalækur | KAL | 9 | 5 |  |  |  |
| Krossá | KRO | 36 | 35 | -34 |  |  |
| Staðará | STA | 31 | 20 | -2 |  |  |
| *Southern* | | | | | | |
| Grenlækur (site 1) | GRE | 45 | 20 | -6 | -1 |  |
| Grenlækur (site 2) | GRE | 23 | 9 |  |  |  |
| Grenlækur (site 3) | GRE | 24 | 16 |  |  |  |
| Þverá á síðu (lindir) | THL | 18 | 18 | -2 | -1 |  |
| Holtsós | HOL | 5 | 4 |  |  |  |
| Húsá | HUS | 4 | 3 |  |  |  |
| *Ölfusá watershed* | | | | | | |
| Hestvatn | HES | 38 | 23 |  | -7 |  |
| Hvítá | HVI | 7 | 3 |  |  |  |
| Ölfusá (site 1) | OLF | 3 | 3 |  |  |  |
| Ölfusá (site 2) | OLF | 7 | 5 |  |  |  |
| Ölfusá (site 3) | OLF | 8 | 8 |  |  |  |
| Sog | SOG | 24 | 23 | -4 |  |  |
| Varmá (Reykjakot) | VAR | 7 | 6 |  | -1 |  |
| Varmá (Vellir) | VAR | 11 | 8 |  |  |  |
| **Headwater populations** | | | | | | |
| *Northern* | | | | | | |
| Mývatn | MYV | 31 | 31 | -20 |  |  |
| Forvöð | FOR | 33 | 22 | -2 | -3 |  |
| *Þingvallavatn watershed* | | | | | | |
| Efra Sog | EFR | 15 | 13 | -8 |  |  |
| Ölfusvatnsá (site D) | FUS | 12 | 7 | -3 |  |  |
| Ölfusvatnsá (site 1) | FUS | 16 | 14 | -7 |  |  |
| Ölfusvatnsá (site 2) | FUS | 11 | 11 | -5 |  |  |
| Ölfusvatnsá (site 3) | FUS | 5 | 5 | -3 |  |  |
| Öxará (Prestakrókur) | OXA | 19 | 19 |  |  |  |
| Öxará (Valhallarbrú) | OXA | 12 | 12 |  |  |  |
| Úlfljótsvatn | ULF | 32 | 25 | -6 | -6 |  |
| Villingavatnsá | VIL | 11 | 11 | -3 |  |  |
| Þingvallavatn (Ólafsdráttur) | THI | 13 | 3 |  |  |  |
| Þingvallavatn (Svínanes) | THI | 47 | 22 | -1 | -2 |  |
| *Hengill mountain* | | | | | | |
| Þverá | THV | 36 | 33 | -18 | -1 |  |
| Hengladalsá (Innstidalur) | HIN | 6 | 6 | -3 |  |  |
| Hengladalsá – Miðdalur (stream 14) | MID | 2 | 2 |  | -1 |  |
| Hengladalsá – Miðdalur (stream 8) | MID | 18 | 13 | -6 |  |  |
| Hengladalsá – Miðdalur (stream 6) | MID | 5 | 3 | -1 |  |  |
| Hengladalsá – Miðdalur (stream 5) | MID | 18 | 18 | -4 |  |  |
| Hengladalsá – Miðdalur (stream 1) | MID | 27 | 18 | -5 |  |  |
| Hengladalsá – Fremstidalur (stream 12) | FRE | 16 | 15 |  | -1 |  |
| *Veiðivötn system* | | | | | | |
| Grænavatn | GRA | 32 | 20 | -4 |  |  |
| Litlisjór | LIS | 67 | 57 | -6 | -3 |  |
| Litla Fossvatn (site 1) | STF | 9 | 9 | -1 |  |  |
| Litla Fossvatn (site 2) | STF | 5 | 5 |  |  |  |
| Litla Fossvatn (site 3) | STF | 5 | 5 | -3 |  |  |
| Stóra Fossvatn | STF | 41 | 17 |  |  |  |
| Stóra Hraunvatn | STH | 36 | 30 | -3 | -5 |  |
| *Other* | | | | | | |
| Leirvogsvatn | LEI | 65 | 18 | -2 | -9 |  |
| Þórisvatn (Austurbotn) | THO | 13 | 9 |  |  |  |
| Þórisvatn (Grasatangi) | THO | 6 | 6 |  |  |  |
| **Reference group** | | | | | | |
| Loch Slapin (Isle of Skye, Scotland) | SLP | 64 | 51 | -4 | -5 |  |

**Table S5**: Mean water temperature (May-October average), standard deviation, number of years sampled, and source for each of the seven sites used in the LFMM analysis. MFRI: Marine and Freshwater Research Institute of Iceland.

| **Site code** | **Site name** | **Region** | **Mean temperature (°C)** | **Years sampled** | **Source** |
| --- | --- | --- | --- | --- | --- |
| STF | Stóra Fossvatn | Highlands | 6.5 | 2007-2019 | MFRI |
| LIS | Litlisjór | Highlands | 7.1 | 2012-2018 | MFRI |
| THI | Þingvallavatn | South-west | 8 | 2008-2019 | MFRI |
| SOG | Sog | South-west | 8.7 | 2008-2019 | MFRI |
| HIN | Hengladalsá (Innstidalur) | South-west (Hengladalir) | 11.8 | 15MAY2006-15OCT2006 | Ólafsson 2019 |
| VAR | Varmá | South-west (Hengladalir) | 13.2 | 2012-2018 | MFRI |
| MID | Hengladalsá (Miðdalur) | South-west (Hengladalir) | 15.7 | 15MAY2006-15OCT2006 | Ólafsson 2019 |

**Table S6**: Genetic diversity parameters for each of the 40 locations sampled, indicated by their population code and grouped by geographic location. Code: the three-letter codes follow Table S1; N: sample size; H_O_: observed heterozygosity; H_E_: expected heterozygosity; AR: allelic richness; Π: nucleotide diversity. Underlined H_O_ and H_E_ values indicate significantly different values (p < 0.05) and the resulting effect is indicated in the last column.

| **Code** | **N** | **H_O_** | **H_E_** | **A_R_** | **Π (x 10^-4^)** | **Heterozygosity** |
| --- | --- | --- | --- | --- | --- | --- |
| **Putative anadromous populations** | | | | | |  |
| *Northern* | | | | | |  |
| KEL | 4 | 0.417 | 0.421 | 1.335 | 4.983 |  |
| SKA | 8 | 0.340 | 0.362 | 1.355 | 5.263 | deficiency |
| SVI | 9 | 0.355 | 0.354 | 1.391 | 6.343 |  |
| *Westfjords* | | | | | |  |
| BAK | 9 | 0.345 | 0.350 | 1.416 | 6.703 |  |
| DUF | 8 | 0.337 | 0.336 | 1.395 | 6.294 |  |
| NOR | 6 | 0.414 | 0.374 | 1.355 | 6.117 | excess |
| NUP | 8 | 0.383 | 0.377 | 1.316 | 5.182 |  |
| SAN | 4 | 0.454 | 0.434 | 1.313 | 4.948 | excess |
| SUN | 8 | 0.356 | 0.349 | 1.386 | 6.276 |  |
| *Western* | | | | | |  |
| HIT | 9 | 0.328 | 0.348 | 1.453 | 6.910 | deficiency |
| KAL | 5 | 0.412 | 0.390 | 1.391 | 6.475 |  |
| STA | 18 | 0.357 | 0.376 | 1.870 | 6.919 | deficiency |
| *Southern* | | | | | |  |
| GRE | 38 | 0.368 | 0.362 | 1.452 | 7.269 |  |
| THL | 15 | 0.397 | 0.396 | 1.443 | 7.104 |  |
| HOL | 4 | 0.404 | 0.416 | 1.451 | 6.445 | deficiency |
| HUS | 3 | 0.468 | 0.471 | 1.316 | 4.673 |  |
| *Ölfusá watershed* | | | | | |  |
| HES | 16 | 0.366 | 0.382 | 1.428 | 6.218 | deficiency |
| HVI | 3 | 0.504 | 0.463 | 1.502 | 8.044 | excess |
| OLF | 16 | 0.367 | 0.375 | 1.502 | 7.669 |  |
| SOG | 19 | 0.366 | 0.372 | 1.464 | 7.322 |  |
| VAR | 13 | 0.434 | 0.421 | 1.428 | 6.818 | excess |
| **Head-water populations** | | | | | |  |
| *Northern* | | | | | |  |
| MYV | 11 | 0.476 | 0.475 | 1.254 | 3.934 |  |
| FOR | 17 | 0.389 | 0.397 | 1.229 | 3.569 |  |
| *Þingvallavatn watershed* | | | | | |  |
| EFR | 5 | 0.394 | 0.389 | 1.210 | 3.342 |  |
| FUS | 19 | 0.357 | 0.377 | 1.232 | 3.555 | deficiency |
| OXA | 31 | 0.391 | 0.399 | 1.213 | 3.435 |  |
| ULF | 13 | 0.443 | 0.431 | 1.239 | 3.832 |  |
| VIL | 8 | 0.372 | 0.362 | 1.219 | 3.587 |  |
| THI | 22 | 0.407 | 0.409 | 1.240 | 3.658 |  |
| *Hengill mountain* | | | | | |  |
| THV | 14 | 0.418 | 0.433 | 1.132 | 1.981 |  |
| HIN | 3 | 0.509 | 0.454 | 1.276 | 4.677 | excess |
| MID | 37 | 0.370 | 0.384 | 1.385 | 5.823 | deficiency |
| FRE | 14 | 0.418 | 0.415 | 1.386 | 6.173 |  |
| *Veiðivötn system* | | | | | |  |
| GRA | 16 | 0.387 | 0.389 | 1.397 | 6.374 |  |
| STF | 32 | 0.396 | 0.397 | 1.271 | 4.378 |  |
| LIS | 48 | 0.370 | 0.388 | 1.417 | 6.674 | deficiency |
| STH | 22 | 0.426 | 0.408 | 1.399 | 6.488 | excess |
| *Other* | | | | | |  |
| LEI | 7 | 0.347 | 0.374 | 1.147 | 2.193 | deficiency |
| THO | 15 | 0.400 | 0.398 | 1.421 | 6.778 |  |
| **Reference group** | | | | | |  |
| SLP | 42 | 0.382 | 0.393 | 1.487 | 7.329 | deficiency |

**Table S7**: Markers significantly associating with temperature (FDR < 0.001) in eight Icelandic populations of brown trout. The genomic position of SNP’s, p-value, gene ID and product (if in a coding region) are given for each of the 40 SNP’s. The ID and corresponding product of genes located within 5,000 bp are also shown. Selected markers with high p-values (indicated with an asterisk) were used to plot allele frequencies in Figure 5. When more than one marker was present in the same chromosome, the marker with the highest p-value was chosen.

|  |  |  |  |  | **± 5,000 bp** | |
| --- | --- | --- | --- | --- | --- | --- |
| **Chr.** | **BP** | **p-value** | **Gene ID** | **Product** | **Gene ID** | **Product** |
| 2 | 1468624 | 3.63E-07 | N/A | N/A | N/A | N/A |
| 2 | 5092671 | 1.70E-08* | 115149364 | zinc finger CCHC domain-containing protein 2-like | N/A | N/A |
| 4 | 18867263 | 2.14E-08 | 115191614 | uncharacterised mRNA | N/A | N/A |
| 8 | 2637597 | 2.51E-09* | N/A | N/A | 115198050 | uncharacterised transcript variant X2 |
|  |  |  |  |  | 115199264 | U6 spliceosomal RNA (snRNA) |
| 9 | 13411731 | 2.34E-07 | N/A | N/A | N/A | N/A |
| 9 | 13988690 | 5.62E-07 | 115199955 | ral guanine nucleotide dissociation stimulator-like, transcript variant | N/A | N/A |
| 9 | 18976051 | 1.15E-09* | 115200108 | ras-related protein Rab-7a-like | 115200109 | ubiquitin-like protein 4A-B, transcript variant X1 |
|  |  |  |  |  | 115200107 | dynamin-1-like protein, transcript variant X2 |
| 9 | 31582072 | 7.24E-07 | N/A | N/A | 115200404 | protein patched homolog 1-like, transcript variant X1 |
| 10 | 10379767 | 2.19E-09* | 115201088 | arf-GAP with dual PH domain-containing protein 1-like | 115201089 | uncharacterised nc RNA |
| 12 | 75232836 | 6.31E-07 | 115204155 | leucine-rich repeat transmembrane neuronal protein 4-like | N/A | N/A |
| 12 | 75585597 | 3.80E-07 | 115204161 | protein unc-13 homolog B-like transcript variant X2 | N/A | N/A |
| 12 | 77558869 | 1.78E-07 | N/A | N/A | N/A | N/A |
| 14 | 42346626 | 7.24E-07 | N/A | N/A | N/A | N/A |
| 15 | 15900100 | 7.59E-08 | 115148550 | long-chain-fatty-acid-CoA ligase 6-like, transcript variant X2 | 115148551 | interferon regulatory factor 1-like |
| 15 | 28134009 | 5.75E-09 | 115149639 | ubiquitin carboxyl-terminal hydrolase 28-like | N/A | N/A |
| 15 | 29040567 | 6.92E-07 | 115148754 | B-cell CLL/lymphoma 7 protein family member B-A-like | 115148753 | transducin beta-like protein 2 |
|  |  |  |  |  | 115148756 | uncharacterised transcript variant X2 (mRNA) |
| 15 | 54507522 | 2.09E-11* | N/A | N/A | N/A | N/A |
| 16 | 45395128 | 2.04E-08* | N/A | N/A | N/A | N/A |
| 18 | 49919625 | 5.62E-07 | 115153446 | CUB and sushi domain-containing protein 1-like transcript variant X1 | N/A | N/A |
| 19 | 40737603 | 3.63E-08 | N/A | N/A | 115154589 | protein phosphatase 1D-like, transcript variant X4 |
| 20 | 1985556 | 3.09E-07 | N/A | N/A | 115156261 | thrombospondin type laminin G domain and EAR repeats |
| 20 | 54471871 | 2.51E-11* | N/A | N/A | N/A |  |
| 21 | 9422900 | 1.29E-11* | 115156620 | low-density lipoprotein receptor-related protein 8-like transcript | 115156622 | KN motif and ankyrin repeat domain-containing protein 4-like |
| 21 | 12752540 | 3.89E-08 | 115156713 | NOP2/Sun RNA methyltransferase 4 | 115156713 | N/A |
| 22 | 36491658 | 6.92E-07 | 115158663 | rhophilin-1-like | 115158663 | N/A |
| 23 | 26546740 | 5.37E-07 | 115159677 | netrin receptor UNC5D-like transcript variant X1 | 115159677 | N/A |
| 29 | 14575725 | 2.45E-10* | 115166986 | protein unc-13 homolog C-like, transcript variant X5 | 115166986 | N/A |
| 30 | 16509197 | 4.07E-08 | 115168120 | microphthalmia-associated transcription factor-like, transcript variant | 115168120 | N/A |
| 31 | 29097177 | 4.68E-07 | 115169891 | cytochrome c-type heme lyase-like | 115169890 | transmembrane protein 56-B-like transcript variant X3 |
| 31 | 39285782 | 5.50E-07 | N/A | N/A | N/A | N/A |
| 31 | 39729726 | 2.40E-08 | N/A | N/A | N/A | N/A |
| 33 | 23514174 | 1.70E-07 | N/A | N/A | N/A | N/A |
| 33 | 30581198 | 9.33E-09 | N/A | N/A | 115172824 | disintegrin and metalloproteinase domain-containing protein |
| 33 | 30628475 | 2.14E-11 | N/A | N/A | N/A | N/A |
| 33 | 31647100 | 1.15E-09 | N/A | N/A | N/A | N/A |
| 33 | 31647732 | 1.62E-09 | N/A | N/A | N/A | N/A |
| 33 | 31721993 | 2.82E-13* | N/A | N/A | N/A | N/A |
| 35 | 37864866 | 6.92E-08 | N/A | N/A | N/A | N/A |
| 39 | 18838542 | 2.88E-07 | 115179311 | immunoglobulin superfamily member 10-like | N/A | N/A |
| 40 | 15987096 | 2.14E-08 | N/A | N/A | 115180607 | lipase maturation factor 2-like |
|  |  |  |  |  | 115180201 | transmembrane protease serine 9-like |

**Table S8**: Markers with F_ST_ values in the top 0.5% of the distribution along the *S. trutta* genome, reflecting the differences between selected populations of two contrasting habitats (*i.e.* lake vs river). The genomic position of SNP’s, F_ST_ value, gene ID and product (if in a coding region) are given for each of the 22 SNP’s. The ID and corresponding product of genes located within 5,000 bp are also shown.

|  |  |  |  |  | **± 5,000 bp** | |
| --- | --- | --- | --- | --- | --- | --- |
| **Chr.** | **BP** | **F_ST_** | **Gene ID** | **Product** | **Gene ID** | **Product** |
| 1 | 47295799 | 0.314 | 115197615 | integrin beta-3-like transcript variant X3 | 115197615 | integrin beta-3-like transcript variant X3 |
| 3 | 59337017 | 0.292 | N/A | N/A | 115181529 | actin-binding Rho-activating protein-like transcript |
| 6 | 48286865 | 0.315 | N/A | N/A | N/A | N/A |
| 9 | 6089780 | 0.296 | N/A | N/A | N/A | N/A |
| 9 | 6112170 | 0.308 | N/A | N/A | N/A | N/A |
| 9 | 6207269 | 0.291 | 115199764 | ras GTPase-activating protein 1-like transcript variant X2 | 115199764 | ras GTPase-activating protein 1-like transcript variant X2 |
| 16 | 57063161 | 0.344 | 115151090 | translocase of outer mitochondrial membrane 34 | 115151090 | translocase of outer mitochondrial membrane 34 |
| 17 | 42879906 | 0.300 | 115152223 | FYVE RhoGEF and PH domain-containing protein 4-like | 115152223 | FYVE RhoGEF and PH domain-containing protein 4-like |
| 18 | 54821157 | 0.310 | N/A | N/A | N/A | N/A |
| 19 | 21305264 | 0.297 | 115154167 | opioid-binding protein/cell adhesion molecule-like transcript | 115154167 | opioid-binding protein/cell adhesion molecule-like transcript |
| 25 | 11115228 | 0.343 | 115162014 | dystrobrevin beta-like transcript variant X3 | 115162014 | dystrobrevin beta-like transcript variant X3 |
| 25 | 25831499 | 0.302 | N/A | N/A | N/A | N/A |
| 25 | 33874416 | 0.335 | 115162467 | AT-rich interactive domain-containing protein 1B-like | 115162467 | AT-rich interactive domain-containing protein 1B-like |
| 30 | 5472922 | 0.347 | 115168980 | ubiquitin conjugation factor E4 B-like transcript variant X2 | 115168980 | ubiquitin conjugation factor E4 B-like transcript variant X2 |
| 33 | 32443834 | 0.343 | 115172154 | interphotoreceptor matrix proteoglycan 1-like | 115172154 | interphotoreceptor matrix proteoglycan 1-like |
| 33 | 38313996 | 0.316 | N/A | N/A | N/A | N/A |
| 35 | 13472168 | 0.316 | 115174649 | protein Z-dependent protease inhibitor-like | 115174649 | protein Z-dependent protease inhibitor-like |
| 36 | 451587 | 0.310 | 115176234 | kelch-like protein 38 | 115176234 | kelch-like protein 38 |
| 37 | 13571231 | 0.297 | 115176640 | dual specificity tyrosine-phosphorylation-regulated kinase | 115176640 | dual specificity tyrosine-phosphorylation-regulated kinase |
| 41 | 573562 | 0.353 | N/A | N/A | N/A | N/A |
| 41 | 1033739 | 0.361 | N/A | N/A | N/A | N/A |
| 41 | 1034223 | 0.356 | N/A | N/A | N/A | N/A |

**Table S9**: Overlapping SNP’s between population comparisons, shown as the Intersection Size, using Sog as the reference putative anadromous population with special emphasis on Hestvatn.

|  | **Overlap** | | | | |  |  |
| --- | --- | --- | --- | --- | --- | --- | --- |
| **Lake** | **Unique** | **Single** | **Double** | **Triple** | **Quadruple** | **Total** | **% Unique** |
| OXA | 141 | 59 | 11 | 1 | 2 | 214 | 65.9 |
| STF | 101 | 40 | 11 | 1 | 2 | 155 | 65.2 |
| MYV | 80 | 41 | 8 | 1 | 2 | 132 | 60.6 |
| SVI | 28 | 19 | 4 | 0 | 2 | 53 | 52.8 |
| HES | 2 | 3 | 2 | 1 | 2 | 10 | 20.0 |
| **Total** | 352 | 162 | 36 | 4 | 10 | 564 |  |

**Table S10**: Markers present in most of the comparisons between five lakes (*i.e.,* spawning fish from Þingvallavatn at Öxará, Stóra Fossvatn, Mývatn, Svínavatn and Hestvatn) to anadromous brown trout from Sog, shown in Figure 6. The genomic position of SNP’s, gene ID and product (if in a coding region) are given for each of the 15 SNP’s. The ID and corresponding product of genes located within 5,000 bp are also shown.

|  |  |  |  |  | |  | | **± 5,000 bp** | | |
| --- | --- | --- | --- | --- | --- | --- | --- | --- | --- | --- |
| **N° of comparisons** | **Chr.** | **BP** | **Gene ID** | | **Product** | | **Gene ID** | | **Product** |  |
| 5 | 10 | 28586393 | 115201528 | | srbd1 - S1 RNA binding domain 1 | | N/A | | N/A |  |
|  | 25 | 25831499 | N/A | | N/A | | N/A | | N/A |  |
| 4 | 7 | 22010091 | 115197137 | | coro2bb - coronin, actin binding protein, 2Bb | | N/A | | N/A |  |
| 3 | 2 | 32852905 | N/A | | N/A | | N/A | | N/A |  |
|  | 3 | 36387368 | 115174559 | | dual specificity protein kinase CLK4-like | | N/A | | N/A |  |
|  | 5 | 36658707 | 115194104 | | protocadherin-7-like | | N/A | | N/A |  |
|  | 8 | 5260841 | 115198096 | | arf-GAP with Rho-GAP domain, ANK repeat, PH domain- protein | | 115198098 | | uncharacterized/ncRNA |  |
|  | 13 | 64385521 | 115206309 | | calcium-binding protein 8-like | | N/A | | N/A |  |
|  | 14 | 51130646 | 115208349 | | stathmin-3-like | | N/A | | N/A |  |
|  | 18 | 39567488 | 115152676 | | probable E3 ubiquitin-protein ligase HECTD2/pseudo | | N/A | | N/A |  |
|  | 26 | 20624823 | N/A | | N/A | | 115163374 | | claudin-3-like |  |
|  | 27 | 22238557 | 115164601 | | solute carrier family 15 member 4-like | | N/A | | N/A |  |
|  | 31 | 12358347 | 115169560 | | mib1 - MIB E3 ubiquitin protein ligase 1 | | N/A | | N/A |  |
|  | 34 | 23677206 | 115173842 | | trafficking kinesin-binding protein 1-like | | N/A | | N/A |  |
|  | 36 | 2352301 | 115176279 | | angpt2b - angiopoietin 2b | | N/A | | N/A |  |
