# Supplementary material for: Genetic Structure and Signs of Local Adaptation in Icelandic Brown Trout Populations
Source: Ecol Evol. 2026 Sep 11;16(9):e74274. doi: 10.1002/ece3.74274 (PMC13564247; doi:10.1002/ece3.74274)
Supplement: Supplementary file 2 [file ECE3-16-e74274-s002.docx]

**Figure S1**: Properly paired reads mapped onto the *Salmo trutta* genome (x-axis) vs properly paired reads mapped onto the *Salmo salar* genome (y-axis). Three individuals stand out as showing a higher number of paired reads onto the *S. salar* genome. Bak012 (8.58% more reads on *S. salar*) was removed during the data filtering steps due to a high degree of missing data. Bak006 and Duf020 (0.66% and 1.17% more reads on *S. salar*, respectively) were retained in the dataset and did not seem to affect the data analyses.

**Figure S2**: Principal component analysis plot showing individuals along the first six components that explained 33% of the data variation. Two sets of headwater trout separated very strongly but the Scottish reference group only separated in PC4. **Panel A**: PC1 = 12% and PC2 = 8.8%. **Panel B**: PC3 = 4.5% and PC4 = 3.1%. **Panel C**: PC5 = 2.5% and PC6 = 2.1%. Locations were grouped by geographic area and color coded accordingly. Northern sites are shown in magenta hues, Westfjords locations in orange tones, Western ones in teal, Southern in dark blue, Ölfusá-Hvítá watershed locations in light blue and dark blue, and trout from the SW-slopes of the Hengill mountain Varmá in purple hues (Hengladalsá). Þingvallavatn watershed locations in green tones, Veiðivötn and Þórisvatn in red, Leirvogsvatn in pink, and the Scottish reference group in grey.

**Figure S3**: Average pairwise *F_ST_* scores for all locations plotted in a heatmap. Sites were grouped by geographic location/watershed, starting with the Varmá river in the south to two landlocked populations in the North-east and the reference group from Loch Slapin in Scotland. A reference to the three-letter code for each location can be found in Table S1. The colours of each location match those of Figure 4 and Figure S5. *F_ST_* was estimated from Weir and Cockerham‘s formula using the R package *assigner* (Gosselin et al. 2020).

**Figure S4**: Cross-entropy criterion for ancestral population estimation values (K) between 15-23 generated with the *R* package *LEA* on 2946 markers from 600 Brown trout individuals from 58 sampling locations.

**Figure S5**: Ancestry matrix generated with K = 15-17. Note that not all the samples from each location are grouped in the same clusters. At K = 16, Svínavatn collapses into a group containing western locations and at K = 15 the two Westfjords clusters merge into one.

**Figure S6**: Ancestry matrix generated with K = 18-20. Note that not all the samples from each location are grouped in the same clusters. From K = 20 to K = 19 two subgroups from the Scottish reference group Slapin collapse into one and samples from Skjálftavatn join the bigger cluster of Forvöð individuals. From K = 19 to K = 18 a third cluster from the Veiðivötn system disappears as well as a sub cluster containing samples from Kálfalækur.

**Figure S7**: Ancestry matrix generated with K = 21-23. Note that not all the samples from each location are grouped in the same cluster. Moving from K = 23 to K = 22 a third cluster from the Scottish reference group Slapin disappears. From K = 22 to K = 21 a subgroup of Westfjords samples, mostly from Bakkadalsá, joins another Westfjords cluster. A sub cluster of Ölfusá samples is absorbed in the Sog cluster, and the Loch Slapin cluster is subdivided in two groups.

**Figure S8**: Ancestry composition of each fish when K = 17 grouped by location.

**Figure S9**: Histogram of test significance values (p-values) after adjusting with a genomic inflation factor (lambda parameter) = 0.45. This parameter is the median of squared z-scores divided by the median of the χ^2^ distribution. Neutral loci are expected to show a uniform distribution and loci under selection are expected to display a peak close to zero. Ensuring this (*i.e.,* that the null hypothesis H_O_ of selective neutrality is correct) is a key requisite before applying a false discovery rate algorithm (Francois et al. 2016).

**Figure S10**: Manhattan plot along the *S. trutta* genome for markers tested for association with temperature in eight sampling sites in Iceland (*i.e.,* Stóra Fossvatn, Litlisjór, Þingvallavatn, Sog, Varmá, and in the Hengladalsá river at the Innstidalur, Miðdalur, and Fremstidalur valleys). The markers associated significantly with temperature are shown in red, below the FDR = 0.001 (**Panel A**) and FDR = 0.05 (**Panel B**) (negative log of the p-values is shown on the y-axis). Markers are depicted along linkage groups (chromosomes) along the x-axis and SNP coloring of non-associating markers alternates between grey and blue to demarcate linkage groups. Note “Chromosome 41” is an aggregate of unplaced contigs.

**Figure S11**: Allele frequency for five SNP’s with the strongest association to temperature in selected chromosomes, FDR < 0.001 (Benjamini-Hochberg algorithm), in all locations (association was only tested for 8 locations with environmental data).. The three-letter codes match those in Table S1. **A**: marker in chromosome 33 (p-value = 2.82 x 10^-13^) at no known gene. **B**: marker in chromosome 21 (p-value = 1.29 x 10^-11^) at the coding region of a low-density lipoprotein receptor-related protein. **C**: marker in chromosome 15 (p-value = 2.09 x 10^-11^) at no known gene. **D**: marker in chromosome 20 (p-value = 2.51 x 10^-11^) at no known gene. **E**: marker in chromosome 29 (p-value = 2.45 x 10^-10^) at the coding region of a unc-13 homolog C-like protein.

**Figure S12**: Allele frequency for five SNP’s with the strongest association to temperature in selected chromosomes, FDR < 0.001 (Benjamini-Hochberg algorithm), in all locations. The three-letter codes match those in Table S1. **F**: marker in chromosome 9 (p-value = 1.15 x 10^-9^) at the coding region of ras-related protein Rab-7a-like. **G**: marker in chromosome 10 (p-value = 2.19 x 10^-9^) at the coding region of an arf-GAP with dual PH domain-containing protein. **H**: marker in chromosome 8 (p-value = 2.51 x 10^-9^) at no known gene. **I**: marker in chromosome 2 (p-value = 1.70 x 10^-8^) at the coding region of a zinc finger CCHC domain-containing protein. **J**: marker in chromosome 16 (p-value = 2.04 x 10^-8^) at no known gene.

**Figure S13:** Manhattan plot along the *S. trutta* genome showing F_ST_ values along the genome comparing populations from two contrasting habitat types (*i.e.* lake vs river). Markers that are responsible for the highest 0.5% of the F_ST_ values are shown in red. Linkage groups (chromosomes) are listed along the x-axis and SNP coloring alternates between grey and blue to demarcate them. Note “Chromosome 41” is an aggregate of unplaced contigs.

**Figure S14**: Tests of signficance of overlap of markers found to be associated with differences in habitat in two or more lakes, using the SuperExactTest (Wang et al., 2015). On top of each bar are the numbers of genes found in each union, and the total number of genes in each comparison on the right side. Note that this is not identical to UpSet graphs, as the counts of genes in overlapping two rivers also include genes found in unions of those two rivers and others. Therefore the heigth of the columns differs between this graph and Figure 6.

**Figure S15**: Genome wide divergence between brown trout from five lakes (*i.e.,* Hestvatn, Svínavatn, Mývatn, Stóra Fossvatn, Þingvallavtn spawners at Öxará) and putative anadromous trout (*i.e.,* Loch Slapin, Scotland) shown in Manhattan plots of F_ST_ values along the *S. trutta* genome (**Panels A-E**). This represents the same type of analyses as in Figure 06, but in this case Scotland trout was used as reference point instead of river Sog. Markers with F_ST_ values > 0.5 are highlighted in red. Linkage groups (chromosomes) are listed along the x-axis and SNP coloring alternates between grey and blue to demarcate them. Note “Chromosome 41” is an aggregate of unplaced contigs. **Panel F:** UpSet plot displaying the overlap SNP’s between population comparisons, shown as the Intersection Size, using Loch Slapin as the reference putative anadromous population. The Set Size indicates the number of significant markers for each of the comparisons. HES_SCO: Hestvatn vs Loch Slapin; SCO_SVI: Loch Slapin vs Svínavatn; MYV_SCO: Mývatn vs Loch Slapin; SCO_STF: Loch Slapin vs Stóra Fossvatn; OXA_SCO: Öxará vs Loch Slapin. The three-letter codes match those in Table S1.
